# Meta-analysis: implications of interleukin-28B polymorphisms in spontaneous and treatment-related clearance for patients with hepatitis C

**DOI:** 10.1186/1741-7015-11-6

**Published:** 2013-01-08

**Authors:** María A Jiménez-Sousa, Amanda Fernández-Rodríguez, María Guzmán-Fulgencio, Mónica García-Álvarez, Salvador Resino

**Affiliations:** 1 Unit of HIV/Hepatitis Coinfection, National Center for Microbiology, Health Institute Carlos III (ISCIII), 28220 Majadahonda, Madrid, Spain

**Keywords:** meta-analysis, systematic review, interleukin 28B, HCV, polymorphisms

## Abstract

**Background:**

Since 2009, several studies have identified single-nucleotide polymorphisms (SNPs) near the gene encoding for interleukin (IL)-28 (*IL28B*) that are strongly associated with spontaneous and treatment-induced hepatitis C virus (HCV) clearance. Because this large amount of data includes some inconsistencies, we consider assessment of the global estimate for each SNP to be essential.

**Methods:**

Relevant studies assessing *IL28B *polymorphisms associated with sustained virologic response (SVR) and spontaneous clearance (SC) were identified from a literature search of PubMed up to 9 July, 2012. Studies were eligible studies if they included patients infected with HCV or HCV/HIV, or assessed any SNP located within or near the *IL28B *gene, SVR data available under standard treatment, and/or SC data in patients with acute HCV infection. Pooled odds ratios were estimated by fixed or random effects models when appropriate. Variables such as HCV genotype, ethnicity, and type of co-infection were studied.

**Results:**

Of 282 screened studies, 67 were selected for SVR and 10 for SC. In total, 20,163 patients were studied for SVR and 3,554 for SC. For SVR, we found that all SNPs showed strong associations in patients with HCV genotypes 1 and 4, whereas the pooled ORs were almost three times lower for genotypes 2 and 3 (rs12979860 and rs8099917). Regarding ethnicity, the SNP most associated with SVR was rs12979860 in white patients, whereas in East Asians it seemed to be rs8099917. The most studied SNP (rs12979860) showed similar results for patients co-infected with HCV/HIV, as for those infected with HCV only. Finally, rs12979860 and rs8099917 both appeared to be associated with SC.

**Conclusions:**

*IL28B *polymorphisms influence both the outcome of interferon treatment and the natural clearance of HCV. However we did not identify a universal predictor SNP, as the best genetic markers differed depending on patient ethnicity, genotype, and type of infection. Nevertheless, our results may be useful for more precise treatment decision-making.

## Background

Currently, over 200 million people worldwide are chronically infected with hepatitis C virus (HCV) [1]. HCV is an important cause of acute and chronic hepatitis, with only 20% of patients have spontaneous clearance (SC) of the virus. Unfortunately, around a quarter of all patients will progress to develop cirrhosis. Other complications such as co-infection with HIV are common among patients infected with HCV, because both viruses share the same routes of transmission [2].

To date, the mechanisms underlying HCV infection have not been completely defined. Over the past few decades, several host and viral factors have been found to be associated with differences in HCV clearance or persistence. However, an unexplained variability in treatment outcome still exists, suggesting that the genetic background of the host plays an important role [1].

Nowadays, the standard of care for chronic HCV infection consists of pegylated interferon -α plus ribavirin (PEG-IFN/RBV). However, this treatment produces sustained virologic response (SVR) rates in only about 40 to 50% of patients with HCV genotype 1 and approximately 60% in those infected with genotype 4, whereas over 80% of patients with genotypes 2 or 3 achieve SVR [2]. PEG-IFN/RBV treatment is prolonged and costly, and is associated with dose-limiting side-effects, highlighting the need for accurate prediction of treatment failure.

Recently, three independent groups discovered several strongly correlated single genetic polymorphisms (SNPs) that, together with standard therapy, seem to play a role in clearing the virus [3-5]. These polymorphisms are located close to the *interleukin 28B *(*IL28B*) gene on chromosome 19. *IL28B *encodes IFN-λ3, which induces antiviral activity by itself and through the Janus kinase-signal transducer and activator of transcription (JAK-STAT) complex, which induces IFN-stimulated genes (ISGs) that also have antiviral activity against HCV [6]. Beyond their identification, little is known about the mechanisms involved between these genomic variants and viral clearance. However, it has been shown that unfavorable *IL28B *genetic variations are associated with higher pre-activated levels of ISGs, which could explain the poor response in these patients [7,8].

Interest in this gene has led to a large number of publications showing *IL28B *polymorphisms as having an influence on HCV clearance. However, conflicting conclusions have been reached in some cases, with some studies reporting significant associations, and others reporting no such associations [9-11].

The aim of this study was to elucidate the pooled estimated effect of *IL28B *polymorphisms on PEG-IFN/RBV treatment response and SC, by conducting a meta-analysis of all eligible studies published up to 9 July 2012.

## Methods

### Search strategy and selection criteria

Relevant studies were identified by a literature search of PubMed without imposing study-period restrictions, using the following terms: 'hepatitis C', '*IL28B*', 'SNP', 'spontaneous clearance', 'treatment', 'ribavirin' and 'interferon' (for the complete electronic search strategy, see Additional file 1). The information contained in this report is based on articles published before 9 July 2012 in any language. The meta-analysis was conducted following the guidelines published by Sutton *et al. *[12], and the data are reported in accordance with the Preferred Reporting Items for Systematic Reviews and Meta-Analyses (PRISMA) guidelines [13].

We developed strict inclusion and exclusion criteria before reviewing the studies and extracting the data in order to ensure maximum possible homogeneity between studies. Only articles satisfying the following criteria were further evaluated by two independent reviewers: 1) patients infected with HCV or HCV/HIV; 2) any SNP located within or near the *IL28B *gene; and 3) SVR data available for patients receiving standard treatment or d) SC data available for patients with acute HCV infections. Exclusion criteria were: 1) treatment duration of less than 24 weeks; 2) co-infection with HBV; 3) studies that included patients with organ transplantation; 4) studies with sample sizes of less than 40 subjects; 5) SVR data reported by methods other than PCR; 6) HCV genotypes other than 1, 2, 3 or 4.

To select the candidate studies, we carefully screened the title and abstract of each citation. When articles fulfilled the inclusion criteria, we examined the full text and extracted data from the study. When studies included several subgroups that did not all fulfill the inclusion criteria, we only incorporated into the meta-analysis those subgroups that did meet the inclusion criteria.

### Data extraction

Two investigators independently assessed the selected papers and extracted all data. When data were unclear or required assumptions to be made, another investigator was consulted, so that a consensus could be reached before recording an entry in the database. When more than one paper studying the same cohort was found, only the study with the most extensive cohort was reviewed, excluding the remaining overlapping studies or data (for the extracted data from each study, see Additional file 2). When incongruent data were detected for one particular variable, they were discarded. We only included those data that could be confirmed by their appearance in different parts of the text (for instance in the main text and the tables).

When articles provided data from patients of different ethnicities, HCV genotypes, and/or types of infection (HCV and HCV/HIV), these data were divided into subgroups, with each subgroup identified by a sequential letter. Regarding ethnicity, subjects were grouped into the main racial group of the study population based on their geographical origin or ancestry [14]. Studies with more than 99% of the study population described as Caucasian were considered to be Caucasian. For data presentation, genotypes 1 and 4 were considered to be in the same subgroup because of their similarity in response and the same applied for genotypes 2 and 3 [15].

To compare results between studies on fibrosis, we used 4 to 6 Ishak levels for the scale of fibrosis corresponding to a METAVIR score of 3 to 4. HCV viral load data were collected at pre-treatment.

Only patients who had an absence of detectable serum HCV RNA by PCR 24 weeks after treatment cessation were considered to have achieved SVR. For SC, patients with acute HCV infection who had undetectable levels HCV RNA on repeated examination without prior IFN therapy were considered to have spontaneously resolved the infection.

In an attempt to include the majority of studies and to complete any missing data, we contacted some authors of individual studies.

### Quality appraisal

To evaluate the quality of the included studies, two investigators appraised them independently using a checklist based on the Graphic Appraisal Tool for Epidemiological studies (GATE) [16]. Each item was rated as +1 (well reported and reliable), 0 (unclear, insufficient detail provided) or -1 (poorly reported, not useful or reliable). The overall validity of each study was also rated by a similar system: +1 (most of the quality items were fulfilled), 0 (some criteria were not fulfilled), or -1 (few or none of the items were fulfilled).

### Statistical analysis

We calculated whether all studied SNPs in the present meta-analysis concurred with Hardy-Weinberg equilibrium (HWE) [17] using the χ^2 ^test, with equilibrium considered at *P *> 0.05.

Overall, meta-analyses were performed only when two or more papers studying the same SNP were available. In all analyses, pooled odds ratios (ORs) and 95% confidence intervals (CIs) were calculated (favorable homozygous versus heterozygous plus unfavorable homozygous). The significance of the pooled OR was calculated by the *Z*-test, and was considered significant at *P *< 0.05. A fixed effect model (the traditional Mantel-Haenszel test) was used for homogeneous studies [18]. The heterogeneity of each group of ORs was assessed by the χ^2 ^test, which suggests the presence of heterogeneity when *P *< 0.1. Heterogeneity was quantified with the *I*^2 ^metric, which provides a measure of the degree of inconsistency in the studies' results (*I*^2 ^> 50% indicated considerable heterogeneity). When significant heterogeneity existed, a random-effects model (the DerSimonian and Laird method [19]), was used, and a subgroup meta-analysis and forest plot based on ethnicity, HCV genotype, and co-infection data (HCV or HCV/HIV) were performed to identify the effect modifiers [20]. Moreover, the Galbraith plot was used to detect possible outliers of the heterogeneity, which could have biased the combined estimate. This graphical method allowed those studies that had a strong influence on the pooled results to be checked [21,22]. Trials outside the Galbraith limits were trials where the 95% CI did not contain the pooled estimate.

In addition, when heterogeneity was detected, meta-regression analysis was also performed, with the aim of defining the potential effect of the covariates on spontaneous or treatment-related clearance. The regression coefficient obtained describes how SC or SVR changes with each unit increase in the covariate. Significance of the linearity relationship between SC or SVR and the covariate was identified by the *P*-value; the adjusted R^2 ^term indicates the proportion of between-study variance explained by the included variable. The evaluated variables were as follows: genotype (1/4 versus 2/3), ethnicity (Caucasian versus other, and Caucasian versus East Asian), type of viral infection (HCV mono-infection versus HCV/HIV co-infection), viral load (HCV RNA < 6 log_10 _versus HCV RNA ≥ 6 log_10_), advanced fibrosis F3/F4 (≤ 30% versus > 30%), and prior HCV treatment (naive versus previous HCV treatment failures).

Publication bias was assessed by funnel plot and the Egger linear regression test [23,24], which detects funnel plot asymmetry. When the Egger test reported a *P *< 0.05, publication bias was assumed to exist.

Sensitivity analyses [24] were also conducted to assess the consistency of results and to investigate the influence of one study on the overall meta-analysis. It was carried out by sequential omission of individual studies.

All analyses were performed using Stata software (version 11.0; Stata Corporation, College Station, TX, USA).

## Results

### Studies and data included in the meta-analysis

**Figure 1:**
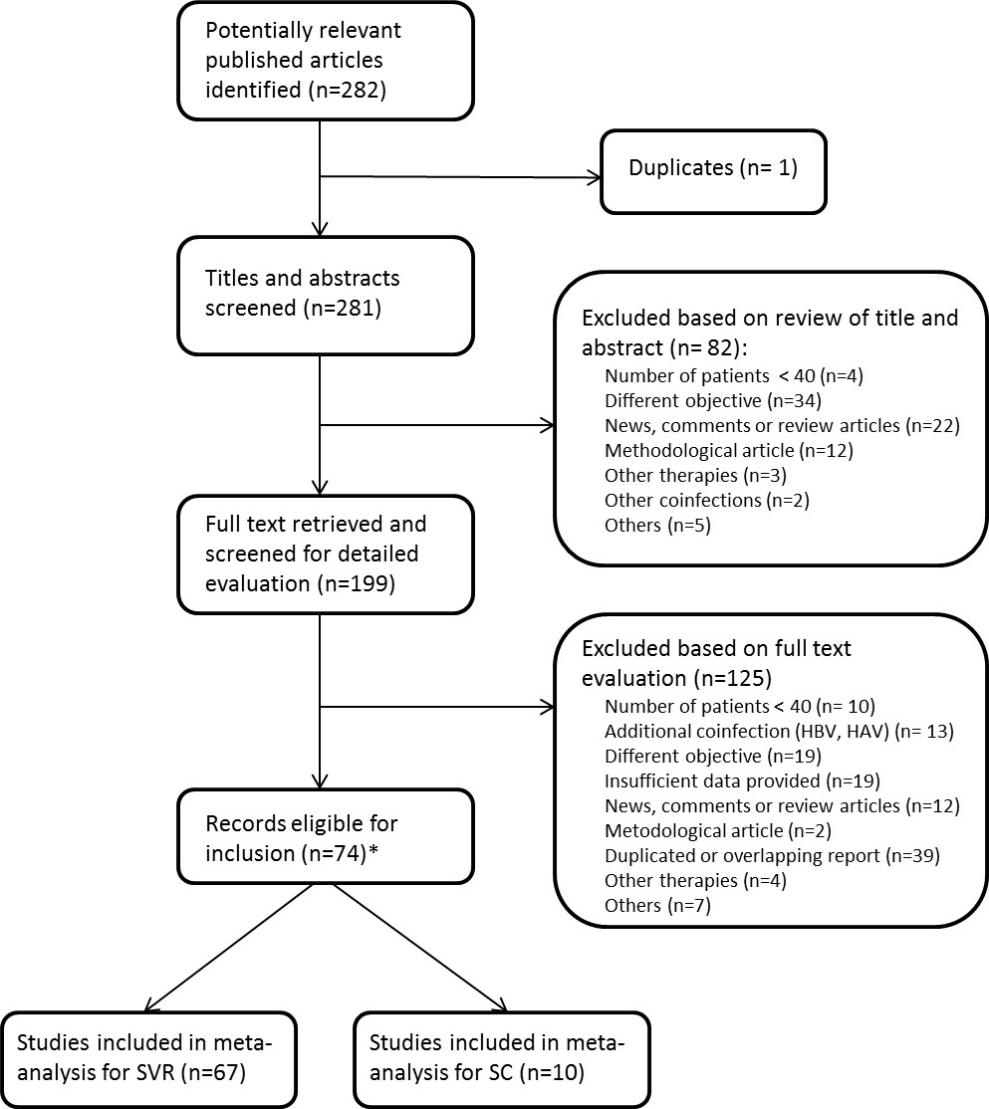
**Flow diagram for the selection of articles for inclusion in the meta-analysis**. *Two studies were potentially eligible for meta-analysis of both sustained virologic response (SVR) and spontaneous clearance (SC).

The literature search identified 282 publications (Figure 1). After initial screening and removal of duplicates (n = 1), 207 articles were rejected based on the title/abstract (n = 82) or the full text (n = 125), because they did not meet the inclusion criteria. This left 74 studies that were eligible for inclusion (see Additional file 2), comprising 67 that were selected for SVR meta-analysis [5,9-11,25-87] and 10 for SC meta-analysis [26,28,65,88-94].; 3 of these studies were included in both analyses [26,28,65]. Regarding the quality appraisal of the included studies for SVR, 21 were rated as +1, 43 as 0, and two as -1 (see Additional file 3), and for SC, four studies were rated as +1 and six as 0 (see Additional file 4).

To date, 21 polymorphisms within or near *IL28B *have been investigated in relation to SVR (rs688187, rs4803219, rs4803221, rs4803223, rs4803224, rs7248668, rs7248931, rs8099917, rs8103142, rs8105790, rs8109886, rs10853727, rs10853728, rs11881222, rs12972991, rs12979860, rs12980275, rs12980602, rs12982533, rs28416813, rs35790907). Those polymorphisms genotyped only in one study had to be discarded from the meta-analysis because of the impossibility of performing statistical analysis. This was the case for two SNPs from Chen *et al. *(2011) [37] (rs28416813 and rs4803219); and ten from Smith *et al. *(2011) [52] (rs12980602, rs12982533, rs35790907, rs4803221, rs4803224, rs688187, rs7248931, rs8109886, rs12972991, and rs10853727). With respect to SC, one SNP from Renda *et al. *(2011) [90] (rs8103142) and five from Rao *et al. *(rs10853728, rs11881222, rs4803219, rs4803223, rs8105790) were excluded. In summary, eight polymorphisms were analyzed in the present study (see Additional file 5). All of the studied SNPs fulfilled the HWE, except for rs12979860, rs11881222, rs8103142, and rs10853728.

The most studied *IL28B *SNPs and their favorable genotypes for SVR were: rs12979860 (CC), rs8099917 (TT), and rs12980275 (AA). The rs12979860 polymorphism was assessed in 12,184 patients from 42 studies; rs8099917 was assessed in 11,839 patients from 39 studies; and rs12980275 was assessed in 2,786 patients from 6 studies. For the SC analysis, 2,340 patients from 7 studies and 1,783 patients from 4 studies were analyzed for rs12979860 and rs8099917, respectively.

### Baseline characteristics

The publication year of the studies ranged from 2009 to 2012 (see Additional file 2 for a summary of the baseline characteristics of the included publications for SVR (n = 67) and SC (n = 10), respectively). The studies were mostly conducted with Caucasian and Asian populations. Nearly 50% of the studies included only patients with genotype 1, whereas patients with genotype 4 were infrequently studied. As regards the type of infection, most of the articles studied patients infected with HCV only, whereas patients co-infected with HCV/HIV were assessed for SVR in only seven articles and for SC in only one article, which was mixed.

When articles were divided into subgroups, each one was identified by a sequential letter (see Additional file 6).

### Publication bias test results

**Figure 2:**
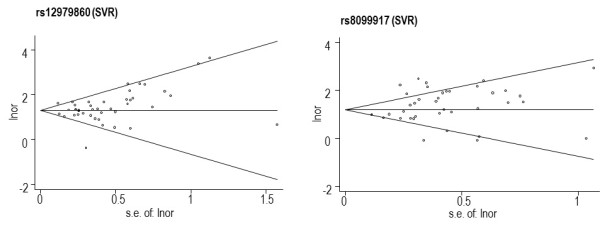
**Publication bias**. Begg's funnel plots with 95% confidence limits, showing publication bias for rs12979860, rs8099917, and rs12980275 for sustained virologic response (SVR) and rs12979860 for spontaneous clearance (SC).

Following recommendations for correct funnel-plot interpretation, SNPs found in less than 10 articles should not be evaluated for publication bias [95], thus, publication bias was only analyzed for rs12979860 and rs8099917 for SVR. Funnel-plot results (Figure 2) and Egger test results (see Additional file 7) showed that there was statistical significance in publication bias only for rs8099917 (*P *= 0.005) in the SVR meta-analysis.

### Overall and subgroup meta-analysis

We performed two overall meta-analyses separately for SVR and SC, with all data grouped by polymorphism. When heterogeneity was identified, we stratified the data into subgroups according to ethnicity (African, African American, Asian, Caucasian, Hispanic, and North African), HCV genotype (1/4, 2/3, and mix) and type of viral infection (HCV or HCV/HIV) (Table 1). In parallel, we performed meta-regression analysis to investigate the possible influence of several variables on the heterogeneity. Genotype and allele frequencies from all analyzed *IL28B *SNPs were also stratified by ethnicity (see Additional file 8).

**Table 1:** Summary of Forest plots showing the associations between *IL28B *polymorphisms and SVR or SC.

Stratification	Subgroup	No.^a^	**OR (95% CI)**^b^	*P *value	**SVR/favorable genotype**^c^	**SVR/unfavorable genotype**^d^	**Weight, %**^e^	**I^2^, %**^f^	***P*-value**^g^
**rs12979860**									

Overall		42	3.77 (3.25 to 4.37)	< 0.001	3,516/4,951	2,775/7,233	100	52.3	< 0.001

By ethnicity	A	1	3.75 (1.60 to 8.79)	0.002	18/30	28/98	1.75	NA	NA

	AA	2	3.19 (0.99 to 10.29)	0.052	26/59	76/414	3.50	71.1	0.063

	As	7	3.27 (2.21 to 4.84)	< 0.001	1,137/1,565	136/315	12.35	40.4	0.122

	C	37	3.63 (4.01 to 4.37)	< 0.001	2,021/2,846	2,168/5,392	64.92	56.5	< 0.001

	H	2	7.17 (0.56 to 92.34)	0.131	38/54	59/161	2.29	81.5	0.020

	NA	1	1.67 (0.52 to 5.38)	0.393	10/16	22/44	1.14	NA	NA

	Mixed	13	3.95 (2.68 to 5.83)	< 0.001	266/381	286/806	14.35	30.1	0.143

	Overall	63	3.59 (3.10 to 4.15)	< 0.001	3,516/4,951	2,775/7,230	100	48.5	< 0.001

By HCV genotype	1/4	37	4.20 (3.61 to 4.90)	< 0.001	1,728/2,647	1,641/5,106	59.33	27.5	0.065

	2/3	15	1.59 (1.14 to 2.21)	0.006	640/779	537/719	16.59	19.8	0.232

	Mixed	11	3.97 (3.30 to 4.77)	< 0.001	1,130/1,506	577/1,386	24.07	0.0	0.581

	Overall	63	3.58 (3.10 to 4.15)	< 0.001	3,498/4,932	2,755/7,211	100	48.3	< 0.001

By type of infection	HCV	57	3.55 (3.04 to 4.14)	< 0.001	3,390/4,765	2,684/6,949	92.53	51.3	< 0.001

	HCV/HIV	6	4.07 (2.66 to 6.21)	< 0.001	126/186	91/284	7.47	0.0	0.436

	Overall	63	3.59 (3.10 to 4.15)	< 0.001	3,516/4,951	2775/7233	100	48.4	< 0.001

**rs8099917**									

Overall		39	3.86 (3.18 to 4.69)	< 0.001	5,222/8,106	1,298/3,733	100	71.9	< 0.001

By ethnicity	A	1	2.02 (0.88 to 4.65)	0.097	37/90	10/39	1.98	NA	NA

	As	30	4.82 (3.80 to 6.11)	< 0.001	3,548/5,535	477/1740	53.23	60.3	< 0.001

	C	20	2.71 (2.07 to 3.54)	< 0.001	1,314/1,982	624/1491	33.68	50.8	0.005

	H	1	11.25 (3.51 to 36.00)	< 0.001	25/29	25/70	1.40	NA	NA

	Mixed	5	2.27 (1.52 to 3.38)	< 0.001	298/468	162/392	9.71	34.4	0.192

	Overall	57	3.63 (3.04 to 4.34)	< 0.001	5,222/8,104	1,298/3,732	100	64.0	< 0.001

By HCV genotype	1/4	37	4.55 (3.76 to 5.52)	< 0.001	3,157/5,473	815/2971	71.23	60.5	< 0.001

	2/3	16	1.59 (1.22 to 2.08)	0.001	1,362/1,706	405/567	21.20	10.1	0.338

	Mixed	4	3.43 (2.36 to 5.00)	< 0.001	643/850	65/162	7.57	0.0	0.733

	Overall	57	3.59 (3.00 to 4.29)	< 0.001	5,162/8,029	1,285/3,700	100	64.1	< 0.001

By type of infection	HCV	50	3.78 (3.14 to 4.57)	< 0.001	4,948/7,681	1,179/3,443	90.07	65.3	< 0.001

	HCV/HIV	3	3.70 (0.96 to 14.31)	0.058	56/104	11/56	3.04	58.8	0.088

	Mix	4	2.07 (1.10 to 3.92)	0.025	218/321	108/234	6.89	50.3	0.110

	Overall	57	3.63 (3.03 to 4.33)	< 0.001	5,222/8,106	1,298/3,733	100	64.0	< 0.001

**rs12980275**									

Overall		6	3.95 (2.39 to 6.53)	< 0.001	1,047/1,433	575/1,353	100	77.7	< 0.001

By ethnicity	As	2	5.00 (1.65 to 15.11)	0.004	633/836	66/206	34.60	88.9	0.003

	C	4	3.44 (1.38 to 8.57)	0.008	374/543	465/1,045	43.85	67.2	0.027

	H	1	8.84 (2.76 to 28.34)	< 0.001	22/26	28/73	10.30	NA	NA

	Mix	1	1.46 (0.50 to 4.24)	0.484	18/28	16/29	11.24	NA	NA

	Overall	8	3.91 (2.31 to 6.62)	< 0.001	1,047/1,433	575/1,353	100	75.7	< 0.001

By HCV genotype	1/4	4	6.33 (2.64 to 15.16)	< 0.001	457/694	428/1,148	59.27	87.4	< 0.001

	2/3	2	1.78 (0.38 to 8.25)	0.460	58/62	86/97	11.79	27.3	0.241

	Mix	2	2.44 (1.38 to 4.32)	0.002	532/677	61/108	28.93	22.5	0.256

	Overall	8	3.91 (2.31 to 6.62)	< 0.001	1,047/1,433	575/1,353	100	75.7	< 0.001

	**Subgroup**	**No**.^a^	**OR [95% CI]**^b^	***P *value**	**SC/favorable genotype**^c^	**SC/unfavorable genotype**^d^	**Weight, %**^e^	**I^2^, %**^f^	***P*-value**^g^

**rs12979860**									

Overall		7	3.20 (2.03 to 5.05)	< 0.001	496/1,091	248/1230	100	81.8	< 0.001

By ethnicity	As	1	1.31 (0.79 to 2.15)	0.291	43/180	37/191	14.70	NA	NA

	C	6	3.78 (2.60 to 5.50)	< 0.001	453/911	211/1,039	85.30	67.9	0.008

	Overall	7	3.20 (2.03 to 5.05)	< 0.001	496/1,091	248/1,230	100	81.8	< 0.001

By HCV genotype	1	2	5.66 (3.28 to 9.77)	< 0.001	253/410	108/522	30.91	70.3	0.067

	Mix	4	2.34 (1.48 to 3.69)	< 0.001	183/528	113/557	54.73	59.8	0.058

	Unknown	1	2.96 (1.75 to 5.02)	< 0.001	60/153	27/151	14.37	NA	NA

	Overall	7	3.20 (2.03 to 5.05)	< 0.001	496/1,091	248/1,230	100	81.8	< 0.001

**rs8099917**									

Overall		4	3.60 (2.70 to 4.81)	< 0.001	340/1,149	72/634	100	46.6	0.132

Abbreviations: A, African; AA, African American; As, Asian; C, Caucasian; H, Hispanic; NA, North African; ND, no data; RCT, randomized controlled trial; SC, spontaneous clearance; SVR, sustained virologic response.

^a^Number of studies or subgroups included for each analysis.

^b^Pooled odds ratios were calculated by fixed or random effects models when appropriate. The significance of the pooled OR was calculated by the *Z*-test, and was considered significant at *P *< 0.05.

^c^Number of patients with favorable genotype that achieved SVR or SC with respect to the total number of patients showing favorable genotype.

^d^SVR or SC/unfavorable genotype state for the number of patients with unfavorable genotype that achieved SVR or SC with respect to the total number of patients showing unfavorable genotype.

^e^Relative weight of each study's contribution to the analysis.

^f^Represents the proportion of heterogeneity.

^g^*P *< 0.1 suggests presence of heterogeneity.

#### Sustained virologic response

**Figure 3:**
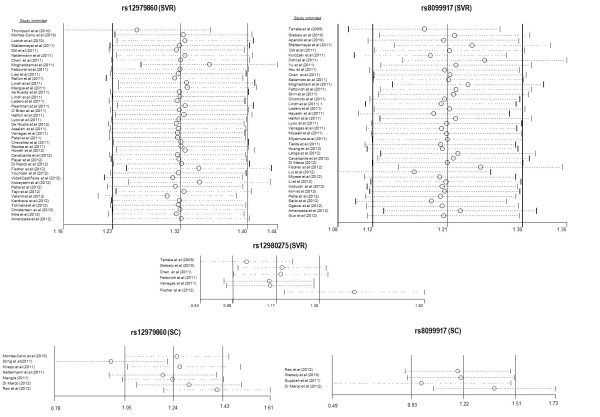
**Sensitivity analysis for rs12979860, rs8099917, and rs12980275 for sustained virologic response (SVR), and rs12979860 for spontaneous clearance (SC)**. Sensitivity analyses were carried out to investigate the influence of any one study on the overall meta-analysis by sequential omission of individual studies.

Sensitivity analysis was carried out using sequential omission of individual studies to investigate the influence of each individual study on the overall meta-analysis (Figure 3).

##### rs12979860

**Figure 4:**
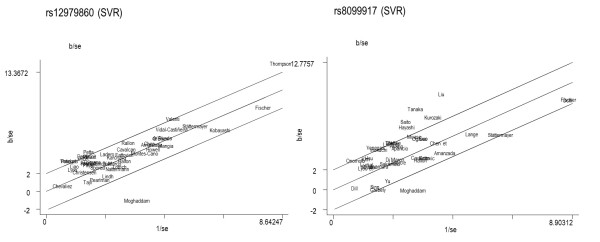
**Galbraith plot**. Detection of studies acting as possible sources of heterogeneity for rs12979860 and rs8099917 for sustained virologic response (SVR). Trials outside the Galbraith limits were trials where the 95% confidence interval did not contain the pooled estimate.

In total, 42 studies reported data for rs12979860 (Table 1; see Additional file 9). The pooled OR for overall data was 3.77 (95% CI = 3.25 to 4.37) and there was heterogeneity (*P *< 0.001; *I*^2 ^= 52.3%). When the Galbraith plot was analyzed, two outliers of heterogeneity were identified (Moghaddam *et al. *[11] and Thompson *et al. *[25]) (Figure 4). A forest plot omitting the mentioned outlier studies was constructed, which reduced heterogeneity (*I*^2 ^= 47.54%), but the significance of the OR was not altered (*P *< 0.001; OR = 3.78; 95% CI = 3.36 to 4.24). In addition, sensitivity analysis showed that none of the 42 studies should be omitted from subsequent statistical analysis. Based on these results and population characteristics, we did not find enough reasons to discard the papers of Moghaddam *et al. *[11] and Thompson *et al. *[25] from subsequent analysis.

When forest plots were constructed based on ethnicity, HCV genotype, and type of viral infection, we obtained the following data:

• Ethnicity: Africans (OR = 3.75; 95% CI = 1.60 to 8.79), African Americans (OR = 3.19;; 95% CI = 0.99 to 10.29), Asians (OR = 3.27; 95% CI = 2.21 to 4.84), Caucasians (OR = 3.63; 95% CI = 4.01 to 4.37), Hispanics (OR = 7.17; 95% CI = 0.56 to 92.34), North Africans (OR = 1.67; 95% CI = 0.52 to 5.38), and mixed (OR = 3.95; 95% CI = 2.68 to 5.83) (see Additional file 10). Note that the subgroup results for Africans (n = 1) [62], African Americans (n = 2) [25,59], Hispanics (n = 2) [9,25], and North Africans (n = 1) [78] are based on analysis of a very small number of studies, therefore there is uncertainty associated with the estimates reported.

• HCV genotype: genotype 1/4 (OR = 4.20; 95% CI = 3.61 to 4.90), genotype 2/3 (OR = 1.59; 95% CI = 1.14 to 2.21), and mixed (OR = 3.97; 95% CI = 3.30 to 4.77) (see Additional file 11). The difference between genotypes 1/4 and 2/3 was significant.

Data for OR (95% CI) were separated by both HCV genotype and ethnicity simultaneously (see Additional file 12). These data can only provide limited insight, because very few articles presented data independently for both variables.

• Type of viral infection: HCV mono-infected (OR = 3.55; 95% CI = 3.04 to 4.14) and HCV/HIV co-infected (OR = 4.07; 95% CI = 2.66 to 6.21) (see Additional file 13).

Subgroup analysis showed that HCV genotype was the only significant cause of heterogeneity between all the studied variables, as the overall analysis was heterogeneous, whereas the subgroup analysis was homogeneous. These data were confirmed by meta-regression, where only the variance between studies attributable to HCV genotype was significant (adjusted *R*^2 ^= 83.61%; *P *< 0.001).

##### rs8099917

There were 39 eligible studies reporting data on rs8099917 (Table 1, see Additional file 14). The pooled OR for overall data was 3.86 (95% CI = 3.18 to 4.69) and the overall heterogeneity was also significant (*P *< 0.001; *I*^2 ^= 71.9%). When the Galbraith plot was constructed (Figure 4), six outliers of the heterogeneity were identified: five studies grouped together (all Asians with genotype 1 and most Japanese patients) and one study (Moghaddam *et al. *[11]), which was also an outlier for the rs12979860 analysis. By excluding these six studies from the analysis, similar pooled OR and significance was obtained (OR = 3.28; 95% CI = 2.81 to 3.83; *P *< 0.001) and heterogeneity was still detected (*I*^2 ^= 56.23%). Likewise, sensitivity analysis showed that none of the 39 studies should be omitted from subsequent statistical analysis.

When forest plots were performed based on ethnicity, HCV genotype, and type of viral infection, we obtained the following data:

• Ethnicity: Africans (OR = 2.02; 95% CI = 0.88 to 4.65), Asians (OR = 4.82; 95% CI = 3.80 to 6.11), Caucasians (OR = 2.71; 95% CI = 2.07 to 3.54), Hispanics (OR = 11.25; 95% CI = 3.51 to 36.00), and mixed (OR = 2.27; 95% CI = 1.52 to 3.38). There was only one publication dealing with a Hispanic population [9], therefore the result relates entirely to this study (Additional file 15). With regard to the Asian and Caucasian subgroups, there was still heterogeneity (*I*^2 ^= 60.3% and 50.8 respectively), which could be due to the mixture of HCV genotypes within each group.

• HCV genotype: genotype 1/4 (OR = 4.55; 95% CI = 3.76 to 5.52), genotype 2/3 (OR = 1.59; 95% CI = 1.22 to 2.08), and mixed (OR = 3.43; 95% CI = 2.36 to 5.00) (see Additional file 16). Again, as was shown for rs12979860, the difference between genotype 1/4 and 2/3 was significant.

• Type of viral infection: HCV mono-infected (OR = 3.78; 95% CI = 3.14 to 4.57), HCV/HIV co-infected (OR = 3.70; 95% CI = 0.96 to 14.31), and mixed (pooled patients with HCV mono-infection, and patients with HCV/HIV co-infection; OR = 2.07; 95% CI = 1.10 to 3.92). It must be taken into account that heterogeneity within the mono-infected subgroup was still high (*I*^2 ^= 65.3%). Regarding HCV/HIV co-infected and mixed subgroups, only a few studies were included (n = 3 and n = 1, respectively), therefore care needs to be taken with the estimates reported (see Additional file 17).

Meta-regression analysis indicated the proportion of significant variance accounting for each different covariate: ethnicity (Caucasians versus Asians: adjusted *R*^2 ^= 26.00%; *P *= 0.002), HCV genotype (adjusted R^2 ^= 51.57%; *P *< 0.001), and advanced fibrosis (adjusted *R*^2 ^= 32.64%; *P *= 0.016). Type of infection and baseline HCV viral load did not seem to influence the heterogeneity (*P *= 0.992 and *P *= 0.087 respectively).

##### rs12980275

The pooled OR for overall data was 3.95; 95% CI = 2.39 to 6.53) and the heterogeneity was significant (*P *< 0.001; I^2 ^= 77.7%) (Table 1, Additional file 18). Galbraith plot identified Tanaka *et al. *[5] as an outlier of the heterogeneity (data not shown); however, the low number of studies prevented us from drawing firm conclusions about this analysis.

Subgroup analysis was performed based on ethnicity, HCV genotype, and type of infection. We obtained the following data:

• Ethnicity: Asians (OR = 5.00; 95% CI = 1.65 to 15.11), Caucasians (OR = 3.44; 95% CI = 1.38 to 8.57), Hispanics (OR = 8.84; 95% CI = 2.76 to 28.34), and mixed (OR = 1.46; 95% CI = 0.50 to 4.24). However, it has to be noted that only two studies were available for the Asian (n = 2 [5,37]) subgroup and only one each for the Hispanic [9] and mixed [28] subgroups (see Additional file 19).

• HCV genotype: genotype 1/4 (OR = 6.33; 95% CI = 2.64 to 15.16), genotype 2/3 (OR = 1.78; 95% CI = 0.38 to 8.25), and mixed (OR = 2.44; 95% CI = 1.38 to 4.32). With respect to the 2/3 and mixed genotype, the data came from only two subgroups and two studies respectively (see Additional file 20).

Regarding type of viral infection, all rs12980275 studies dealt with patients with HCV mono-infection, except the report by Grebely *et al. *[28], in which data from patients with HCV mono-infection and HCV/HIV co-infection were pooled. For this reason, subgroup analyses by type of infection were not carried out.

Using sensitivity analysis (Figure 3), we found that the Fischer *et al. *study [67] on rs12980275 apparently influenced the overall results, and thus a new statistical analysis was performed excluding that paper (data not shown). This influence seemed to affect only genotype 1/4, where heterogeneity was reduced to 0%. Results for genotype 1/4 varied slightly (OR = 9.13; 95% CI = 5.84 to 14.26), although the significance of the OR was not altered. Based on these results and on the population characteristics, we cannot offer any explanation for excluding the Fischer *et al. *study.

##### Other single-nucleotide polymorphisms

Five polymorphisms (rs11881222, rs7248668, rs8103142, rs8105790, rs10853728) were only described in three studies or fewer, leading to less robust meta-analysis results (see Additional files 21 to 25). The pooled OR data were 2.99 (95% CI = 2.31 to 3.87) for rs11881222, 3.87 (95% CI = 2.74 to 5.47) for rs7248668, 2.62 (95% CI = 1.97 to 3.49) for rs8103142, 2.15 (95% CI = 1.61 to 2.86) for rs8105790, and 1.20 (95% CI = 0.82 to 1.76) for rs10853728. There was no heterogeneity except for rs10853728 (*P *= 0.061; *I*^2 ^= 64.3%), but we could not perform heterogeneity analysis by subgroup and/or meta-regression because rs10853728 was genotyped in only three of the studies.

We also recorded all the polymorphisms that were studied in only one study each. Twelve SNPs were reported, with ORs ranging from 1.19 to 9.96; however, only eight of these were significant (rs35790907 (AA), rs12972991 (AA), rs12982533 (TT), rs688187 (GG), rs4803221 (CC), rs8109886 (CC), rs12980602 (TT), and rs4803219 (CC); see Additional file 26).

#### Spontaneous clearance

##### rs12979860

The overall pooled OR was 3.20 (95% CI = 2.03 to 5.05) (Table 1; see Additional file 27). Heterogeneity was detected (*P *< 0.001; *I*^2 ^= 81.8%), therefore, subgroup analysis was performed based on ethnicity and HCV genotype.

• Ethnicity: Asians (OR = 1.31; 95% CI = 0.79 to 2.15) and Caucasians (OR = 3.78; 95% CI = 2.60 to 5.50) (see Additional file 28).

• HCV genotype: genotype 1 (OR = 5.66; 95% CI = 3.28 to 9.77), mixed (OR = 2.34; 95% CI = 1.48 to 3.69) and unknown (OR = 2.96; 95% CI = 1.75 to 5.02) (see Additional file 29).

It must be taken into consideration that the results for Asian patients and for genotype 1 correspond to one and two studies, respectively. For Caucasians, a high level of heterogeneity was detected (*I*^2 ^= 67.9%).

Sensitivity analysis (Figure 3) showed that the study by Dring *et al. *[88] might be influencing the overall statistical analysis. For this reason, we also analyzed the data after removing this study. The results were similar and the significance of the OR was not altered. Based on these results and to the population characteristics, there was no reason to exclude this study.

##### rs8099917

Only four studies reported data for rs8099917 (Table 1; see Additional file 30). Sensitivity analysis showed that none of the four studies should be omitted from subsequent statistical analysis. Pooled OR was 3.60 (95% CI = 2.70 to 4.81), and there was no heterogeneity.

##### rs12980275

Two studies reported very different data for rs12980275, leading to non-significant results (OR = 2.73; 95% CI = 0.33 to 22.38) and high heterogeneity (*I*^2 ^= 81.5%) (Table 1; see Additional file 31).

##### Other single-nucleotide polymorphisms

We also recorded all the polymorphisms for SC that were analyzed in only one study each. In total, seven SNPs were reported, with ORs ranging from 1.19 to 14.88; however, only four of them (rs10853728 (CC), rs12980275 (AA), rs8105790 (TT), rs8103142 (TT)) were significant, although most of them had very high CIs (see Additional file 32).

## Discussion

There is growing evidence that polymorphisms can contribute to differences in complex disease traits between individuals. Since 2009, several studies have shown that there is an important association between *IL28B *polymorphisms and HCV clearance. However, the mechanism of this association remains unclear, and is still under study.

To our knowledge, a total of five reviews containing data from meta-analyses have been published to date about the relationship between *IL28B *polymorphisms and SVR [96-100]. The reports of Romero-Gomez *et al. *and Li *et al. *are limited because they involved literature searches only up to January and May 2010, respectively, leading to the selection of a low number of studies in both cases (only seven studies). In addition, all meta-analyses were performed for rs12979860 and rs8099917 only. The other three meta-analyses were broader, ranging from 17 to 36 studies. However, all of them analyzed only the effect of the *IL28B *polymorphisms rs12979860 and rs8099917 on SVR by ethnicity and HCV genotype, while the study by Scheiber *et al. *was limited to HCV genotypes 2 and 3. In addition, the literature searches of these meta-analyses included publications only up to the end of 2011. Consequently, our meta-analysis provides the most up-to-date compilation of studies, including 74 articles, a larger number of SNPs, and association analysis with other clinical situations such as SC. In addition, this is the first meta-analysis investigating the association between *IL28B *polymorphisms and SC, to our knowledge. Therefore, because there has been a very large increase in the number of papers, especially in 2012, it is necessary to assess all data and elucidate controversial or inconclusive results. This meta-analysis has allowed us to estimate the overall OR of all studies, and functions as a robust tool to investigate discrepant results.

Based on the global analysis, there was a significant association between the favorable genotype of seven studied SNPs (rs12979860, rs8099917, rs12980275, rs8105790, rs11881222, rs8103142, and rs7248668) and SVR. In most of the cases, the probability of achieving SVR in patients with a favorable genotype was more than double that in patients with an unfavorable genotype. Although the majority of results had similarities, the magnitude of the association was different in many cases. This could be caused by the different criteria considered in each individual study. For this reason, our meta-analysis focused on displaying general conclusions about the trend of this association. Moreover, we investigated several variables that might contribute to the different magnitude of associations found in different studies.

### IL28B and ethnicity

The significant genetic association of all *IL28B *SNPs seems to be due to the high linkage disequilibrium (LD) of this genomic region [3-5], which varies across ethnicities. Regarding the Asian population, some authors have described strong LDs between rs12980275, rs8105790, rs11881222, rs8099917, rs7248668, rs10853728, and rs12979860 [5,37]. For Caucasian populations, the results are slightly more diverse depending on the genotyped platform used. Thus, Ge *et al. *[3] reported a strong LD between rs12979860 and rs12980275, whereas Suppiah *et al. *[4] reported that rs12980275 is strictly linked to rs8105790, rs8103142, rs8109886, and rs8099917. Finally, there was a low LD between rs12979860 and rs12980275 for African Americans (r^2 ^= 0.56), whereas for Hispanics, a higher LD (r^2 ^= 0.88) was obtained [3]. Our results have shown that in respect to the favorable *IL28B *genotype frequency of rs12979860, there was a marked differential distribution between racial groups (in order from highest to lowest frequency): Asian, Caucasian, North African, Hispanic, African, and African American. Regarding rs8099917, the order was similar, except for African populations, which showed frequencies that were intermediate between Asians and Caucasians, as shown by Thompson *et al. *[25]. This differential distribution seems to explain much of the observed clinical differences between ethnic groups in response to treatment [101].

We found similar association for rs12979860 for Asians (OR = 3.27) and Caucasians (OR = 3.63). The strength of the association in Asians was almost double that for Caucasians for rs8099917. Similarly, in the case of rs12980275, a larger OR was seen for Asians than for Caucasians. However, rs12980275 was only represented by two studies in Asians and four in Caucasians, therefore the most reliable results and the most robust conclusions were obtained for rs12979860 and rs8099917.

It is also important to note that in our study, the significant association between favorable genotypes and SVR was lost in several instances, such as for rs12979860 in African American and Hispanic patients. However, these data should also be interpreted with caution, because only two studies were included for each ethnicity [9,25,59]. Regarding African Americans, the results were right at the limit of significance (OR = 3.19; *P *= 0.052), which may be related to the low LD described for African Americans. Owing to the scarcity of available results, new studies in these populations are necessary, especially ones that investigate the effects of different SNPs. For Hispanics, both studies individually showed a significant association, but this significance was lost upon performing the meta-analysis. This could be due to the extremely wide confidence interval of the Venegas *et al. *study [9]. On the other hand, rs8099917 and rs12980275 seem to be strongly associated for Hispanic populations; however, these results corresponded to only one study, which prevents us from drawing any firm conclusions.

### *IL28B *and hepatitis C virus genotype

To date, a broad association between favorable *IL28B *genotypes and SVR has been described in patients infected with HCV genotype 1 [50,102], with a similar association described for genotype 4, although this has been less studied. However, conflicting results have been published about HCV genotypes 2/3 [33,41]. One of our goals was to discern the pooled significance of such an association, which would have relevance to the decision of initiating therapy. As expected, we found that the favorable genotypes of polymorphisms rs12979860, rs8099917, and rs12980275 were positively associated with SVR for HCV genotypes 1 and 4. Regarding the HCV genotypes 2 and 3, the polymorphisms rs12979860 and rs8099917 showed significant associations. However, the strength of this association was almost three times lower than for genotypes 1 and 4, and in addition, we found that the Asian population was solely responsible for this association in rs8099917. The generally reduced association for patients with HCV genotypes 2/3 could be related to the high rate of SVR present in these IFN-sensitive genotypes, for which larger sample sizes are required to find significant differences [7]. In summary, our findings show that *IL28B *polymorphisms are a strong pre-treatment predictor for SVR in patients with HCV genotypes 1 and 4, but its usefulness is limited for other genotypes.

### IL28B and type of viral infection

The predictive value of *IL28B *polymorphisms has been extensively studied in patients with HCV mono-infection, but only seven eligible studies included patients with HIV/HCV co-infection [28,29,35,42,61,63,85]. After stratifying by type of infection, we found that in patients co-infected with HIV/HCV, the strength of association between rs12979860 and SVR was similar to that for patients with HCV mono-infection. For rs8099917, only the study by Aparicio *et al. *[29] provided data for patients with HIV/HCV co-infection, which was also divided by HCV genotypes (1, 3, and 4). This study did not show any overall significant association, but when we analyzed the data in more depth, we found differences related to HCV genotype, with only HCV genotype 1 being significantly associated with SVR. Therefore, the benefit of *IL28B *genotyping seems to apply to both patients with HCV genotype 1 mono-infection and co-infection. HIV/HCV co-infection could play a significant role in treatment response, but further studies are necessary to confirm this. Again, results should be interpreted with caution.

### *IL28B *polymorphisms and spontaneous clearance of hepatitis C virus

The identification of markers predicting the persistence of HCV infection is very important to distinguish between patients whose acute hepatitis C resolves and those who develop a chronic hepatitis C infection. Roughly, 20% of patients infected with HCV have SC of the virus. The mechanism of this is not clear, but epidemiological, viral, and host factors have all been associated with the difference in HCV clearance likelihood. Thomas *et al. *[103] showed that rs12979860 strongly enhances the likelihood of clearance of HCV among individuals of either European or African ancestry. However, to date, few articles about the *IL28B *polymorphisms involved in SC have been written. Owing to the low number of published articles, we could perform meta-analyses only for rs12979860 and rs8099917. In both cases, a clear association was detected. These results seem completely plausible because all individual studies analyzed showed significant associations for rs12979860 and rs8099917, except for Asian populations, which were represented by only one study [94]. Stratification was possible only for rs12979860. Regarding ethnicity, significant results were obtained only for Caucasians, with similar pooled OR as for SVR.

With respect to HCV genotype, a strong association was detected for rs12979860 in HCV genotype 1. It has been shown that HCV genotype influences hepatitis C chronicity, as patients infected with genotype 1/4 who harbor favorable *IL28B *genotypes are less likely to have chronic HCV infection. By contrast, this protective effect is not seen for infections with either genotypes 2 or 3 [1]. Because of insufficient data, we could not perform a stratified study on other HCV genotypes or ethnicity. Additional studies on these variables are needed to clarify this association.

### IL28B polymorphisms as clinical predictors

During the past number of years, the main focus in HCV infection has been the identification of markers or factors predicting the likelihood of achieving SVR. Recently, some countries have incorporated *IL28B *genotyping as a diagnostic criterion in clinical practice [7]. In those patients with unfavorable *IL28B *genotypes, which result in response rates of less than 40%, clinicians may consider deferral of treatment until novel therapies are licensed, something likely to occur soon [7]. However, *IL28B *genotype is not solely responsible for therapy response. Indeed, our meta-regression data suggested that several other factors such as ethnicity, HCV genotype, and stage of fibrosis might have a significant effect on SVR and/or SC. Recently, a model including *IL28B *genotype (rs12979860) and four clinical variables (pre-treatment viral load, ratio of alanine and aspartate transaminases, Ishak fibrosis score, and previous treatment with ribavirin) has been developed [51]. This model predicts SVR in patients of European ancestry with HCV genotype 1 who have failed to respond to previous treatment [51]. This algorithm has shown a high predictive ability, but as the authors pointed out, it could be improved in future studies by including other relevant variables such as ethnicity and HCV genotype. Another predictive model has been described for patients with HCV/HIV co-infection, including two host-related variables (the *IL28B *SNP rs12979860 and the level of liver stiffness) and two HCV-related variables (genotype and viral load) [104]. This model was found to have an adequate predictive index, but it could also be enhanced by incorporating HIV variables such as viral load. Apart from the aforementioned variables, it would be of great value for clinical practice if future algorithms could be designed that were applicable to different circumstances, such as naive patients or patients with SC, for instance.

### Future directions

The number of studies on new antiviral therapies have risen in the past few years. Therefore, it would have been interesting to investigate whether *IL28B *polymorphisms also play a predictive role in novel therapies such as direct-acting antivirals (DAAs). However, it was not been possible here because only a few studies have been published that include data on novel therapies. Our literature search returned five studies involving triple therapy. Three of them involved standard of care (PEG-IFN/RBV) with inclusion of telaprevir in the same cohort [105-107], while the fourth studied the inclusion of danoprevir [108], and the fifth studied the inclusion of boceprevir [109]. As we could not perform meta-analysis on these, all of these were excluded. These therapies, which are based on protease inhibitors, are the most advanced DAAs in clinical development. However, any influence of *IL28B *polymorphisms on the outcome of these novel therapies is not clear. These few studies have shown that, regardless of treatment history, *IL28B *SNPs seem to enhance rapid, early, and SVR when combined with PEG-IFN/RBV in patients with chronic HCV genotype 1 infection [109,110]. Further studies are needed to clarify this association.

Finally, to properly interpret our results, some considerations have to be taken into account. Our meta-analysis was performed by using the unadjusted raw data provided from each study, whereas most of the results given by the authors were previously adjusted by age, fibrosis stage, HCV viral load, and/or other factors. For this reason, our ORs may differ slightly from those cited by the original articles. For rs8099917, we identified publication bias, which could indicate that smaller studies dealing with this SNP could have been more likely to be published if their results were significant than if their results were negative or inconclusive. When heterogeneity was evaluated, studies were stratified by ethnicity, genotype, and type of infection, but in some cases heterogeneity remained, indicating the possibility that different causes of heterogeneity may exist. As we have previously mentioned, the number of studies in some subgroup analyses was too small, which led to weak results. As for the five least studied polymorphisms (rs11881222, rs7248668, rs8103142, rs8105790, rs10853728), results are limited and new studies are still needed. Consequently, these results should be interpreted with caution.

## Conclusions

*IL28B *polymorphisms influence both IFN treatment outcomes and the natural clearance of HCV infection. However, although we cannot provide a biological explanation, our findings indicate the most adequate genetic marker seems to vary depending on ethnicity, HCV genotype, and type of viral infection. Taking into account the most robust analyses, the SNP most associated with SVR in Caucasians was rs12979860, whereas in Asians it seemed to be rs8099917. However, for Africans, African Americans, North Africans, and Hispanics, extensive studies are still needed. After analyzing the conflicting results described above for genotypes 2 and 3, our data seem to indicate that rs8099917 is apparently the most adequate predictive marker for SVR with these genotypes. For patients with HCV/HIV co-infection, the most studied SNP (rs12979860) gave similar results to those seen for patients with HCV mono-infection patients. Finally, both rs12979860 and rs8099917 were clearly associated with SC. Moreover, because *IL28B *genotyping needs be performed only once in a patient's life, it is relatively cheap and provides high predictive value. *IL28B *polymorphisms could thus be used to perform personalized treatment in clinical practice, which could play a substantial role in the selection of candidates for standard treatment versus triple therapy with DAAs.

## Abbreviations

CI: confidence interval; DAA: direct-acting antiviral; HCV: Hepatitis C virus; HIV: human immunodeficiency virus; *I*^2^: value that provides a measure of the degree of heterogeneity; *IL28B*: *interleukin 28B *gene; ISGs: interferon-stimulated genes; LD: linkage disequilibrium; OR: odds ratio; PEG-IFN/RBV: pegylated interferon-α plus ribavirin; SC: spontaneous clearance; SNP: single-nucleotide polymorphism; SVR: sustained virologic response.

## Competing interests

The authors declare that they have no competing interests.

## Authors' contributions

MAJS, AF, and SR designed the study. MAJS and AF collected all data, performed the statistical analysis, and drafted the report. MAJS, AFR, MGA, MGF, and SR interpreted the data and critically reviewed the report. All authors have approved the final version of the manuscript.

## Pre-publication history

The pre-publication history for this paper can be accessed here:

http://www.biomedcentral.com/1741-7015/11/6/prepub

## Supplementary Material

Additional file 1 **Figure S1, Search terms**. Relevant studies were identified by a literature search of PubMed without imposing study period restrictions. Click here for file

Additional file 2 **Table S1, Summary of characteristics of all studies included for pegylated interferon-α plus ribavirin (PEG-IFN/RBV) treatment outcome and spontaneous clearance**. * Data belongs to original sample size, not to the genotyped subpopulation. Abbreviations: A, African; AA, African American; As, Asian; C, Caucasian; H, Hispanic; NA, North African; ND, no data; RCT, randomized controlled trial. Click here for file

Additional file 3 **Table S2, Quality appraisal for meta-analysis of sustained virologic response (SVR)**. 1) Source population well described? 2) Population well described and appropriate? 3) Did participants represent those eligible? 4) Was information on previous hepatitis C virus (HCV) treatment information reported? 5) Inclusion/exclusion criteria reported? 6) Type of treatment well described? 7) Outcome measures well described and without incongruencies? 8) Outcome measurement complete? (That is, all genotype counts reported?) 9) Assessment of Hardy-Weinberg equilibrium? 10) Description of what genetic model was assumed? 11) Consideration of genotyping errors and confirmation of results? 12) Information of linkage disequilibrium? 13) Information on haplotypes? 14) Responder and non-responder groups comparison at baseline? 15) Raw data given or calculable? 16) Study sufficiently powered? 17) Statistical methods appropriate? 18) Study results internally valid (that is, unbiased)? (Summary of items 5 to 8 and 14 to 17)? 19) Genetic study reliable? (Summary of items 9 to 13). 20) Results generalizable to the source population (that is, external validity)? (Summary of items 1 to 4). 21) Overall study quality? Abbreviations: NA, not applicable; NR, not reported. Click here for file

Additional file 4 **Table S3, Quality appraisal for SC meta-analysis**. 1) Source population well described? 2) Population well described and appropriate? 3) Did participants represent those eligible? 4)? Was information on previous hepatitis C virus (HCV) reported? 5) Inclusion/exclusion criteria reported? 6) Type of treatment well described? 7) Outcome measures well described and without incongruencies? 8) Outcome measurement complete? (That is, all genotype counts reported?) 9) Assessment of Hardy-Weinberg equilibrium? 1) Description of what genetic model was assumed? 11) Consideration of genotyping errors and confirmation of results? 12) Information on linkage disequilibrium? 13) Information on haplotypes? 14) Spontaneous responder and non-responder groups comparison at baseline? 15? Raw data given or calculable? 16) Study sufficiently powered? 17) Statistical methods appropriate? 18) Study results internally valid (that is, unbiased)? (summary of items 5 to 8 and 14 to 17.) 19) Genetic study reliable? (summary of items 9 to 13.) 20) Results generalizable to the source population (that is, external validity)? (summary of items 1 to 4.) 21) Overall study quality? Abbreviations: NA, not applicable; NR, not reported. Click here for file

Additional file 5 **Figure S2, The location of the studied single-nucleotide polymorphisms (SNPs) in the genome**. * SNPs most studied in recent years. Click here for file

Additional file 6 **Table S4, Identification of all subgroups available for each study**. Click here for file

Additional file 7 **Table S5, Egger test result bias for rs12979860 and rs8099917 for sustained virologic response (SVR)**. No., number of studies; Coef., asymmetry regression coefficient; Std. Err., standard error; t, statistic; P > |t|, significance; and 95% CI, confidence interval. Coef. corresponds to the intercept value in the regression equation, which estimates the asymmetry of the funnel plot. Positive values (Coef. > 0) indicate higher levels of effect size in studies with smaller sample sizes. Click here for file

Additional file 8 **Table S6, Genotype and allele frequencies stratified by ethnicity for all the polymorphisms included in the meta-analysis**. **(a) **Sustained virologic response (SVR) and **(b) **spontaneous clearance (SC).Abbreviations: A, African; AA, African American; As, Asian; C, Caucasian; H, Hispanic; NA, North African; n.d., no data available. Favorable genotypes: rs12979860 (CC), rs8099917 (TT), rs12980275 (AA), rs8105790 (TT), rs11881222 (AA), rs8103142 (TT), rs7248668 (GG). There is conflicting information for rs10853728. Click here for file

Additional file 9 **Figure S3, Overall forest plot showing the association of rs12979860 with sustained virologic response (SVR)**. The vertical continuous line indicates no difference for SVR regarding *IL28B *genotype. The size of each square denotes the proportion of information provided by each trial. Pooled odds ratios were calculated from random-effects models with the DerSimonian-Laird method. **(a) **The number of patients with the favorable genotype (CC) who achieved SVR with respect to the total number of patients having the favorable genotype. **(b) **The number of patients with the unfavorable genotype (CT+TT) who achieved SVR with respect to the total number of patients having the unfavorable genotype. The dashed vertical red line represents overall OR. Click here for file

Additional file 10 **Figure S4, Forest plot showing the association between rs12979860 and sustained virologic response (SVR) stratified by ethnicity**. See description in Figure S3. Click here for file

Additional file 11 **Figure S5, Forest plot showing the association between rs12979860 and sustained virologic response (SVR) stratified by hepatitis C virus (HCV) genotype**. See description in Figure S3. Click here for file

Additional file 12 **Table S7, Subgroup analysis by ethnicity and hepatitis C virus (HCV) genotype (pooled odds ratio and 95% confidence interval)**. ‡Data from only one study. * *P *< 0.05; ** *P *< 0.001. Abbreviations: A, African; AA, African American; As, Asian; C, Caucasian; H, Hispanic; n.d., no data available. Click here for file

Additional file 13 **Figure S6, Forest plot showing the association between rs12979860 and sustained virologic response (SVR), stratified by type of infection: hepatitis C virus (HCV) mono-infection and HCV/HIV co-infection**. For details, see main description in Figure S3. Click here for file

Additional file 14 **Figure S7, Overall forest plot showing the association between rs8099917 and sustained virologic response (SVR)**. Superscripts: number of patients with **(a) **favorable genotype (TT) or **(b) **unfavorable genotype (TG+GG) who achieved SVR, with respect to the total number of patients with the favorable or unfavorable genotype, respectively. For extended details, see main description in Figure S3. Click here for file

Additional file 15 **Figure S8, Forest plot showing the association between rs8099917 and sustained virologic response (SVR), stratified by ethnicity**. Superscripts: number of patients with **(a) **favorable genotype (TT) or **(b) **unfavorable genotype (TG+GG) who achieved SVR, with respect to the total number of patients having the favorable or unfavorable genotype, respectively. For complete details, see main description in Figure S3. Click here for file

Additional file 16 **Figure S9, Forest plot showing the association between rs8099917 and sustained virologic response (SVR) stratified by hepatitis C virus (HCV) genotype**. Superscripts: number of patients with **(a) **favorable genotype (TT) or **(b) **unfavorable genotype (TG+GG) who achieved SVR with respect to the total number of patients having the favorable or unfavorable genotype, respectively. For complete details, see main description in Figure S3. Click here for file

Additional file 17 **Figure S10, Overall forest plot showing the association between rs8099917 and sustained virologic response (SVR) stratified by type of infection: hepatitis C virus (HCV) mono-infection and HCV/HIV co-infection**. Superscripts: number of patients with **(a) **favorable genotype (TT) or **(b) **unfavorable genotype (TG+GG) who achieved SVR, with respect to the total number of patients having the favorable or unfavorable genotype, respectively. For extended details, see main description in Figure S3. Click here for file

Additional file 18 **Figure S11, Overall forest plot showing the association between rs12980275 and sustained virologic response (SVR)**. Superscripts: number of patients with **(a) **favorable genotype (AA) or **(b) **unfavorable genotype (AG+GG) who achieved SVR, with respect to the total number of patients having the favorable or unfavorable genotype, respectively. For extended details, see main description in Figure S3. Click here for file

Additional file 19 **Figure S12, Forest plot showing the association between rs12980275 and sustained virologic response (SVR), stratified by ethnicity**. Superscripts: number of patients with **(a) **favorable genotype (AA) or **(b) **unfavorable genotype (AG+GG) who achieved SVR, with respect to the total number of patients having the favorable or unfavorable genotype, respectively. For extended details, see main description in Figure S3. Click here for file

Additional file 20 **Figure S13, Forest plot showing the association between rs12980275 and sustained virologic response (SVR) stratified by hepatitis C virus (HCV) genotype**. Superscripts: number of patients with **(a) **favorable genotype (AA) or **(b) **unfavorable genotype (AG+GG) who achieved SVR, with respect to the total number of patients having the favorable or unfavorable genotype, respectively. For extended details, see main description in Figure S3. Click here for file

Additional file 21 **Figure S14, Overall forest plot showing the association between rs11881222 and sustained virologic response (SVR)**. Pooled odds ratios were calculated from fixed-effects models with the Mantel-Haenszel method. Superscripts: number of patients with **(a) **favorable genotype (AA) or **(b) **unfavorable genotype (AG+GG) who achieved SVR, with respect to the total number of patients having the favorable or unfavorable genotype, respectively. For extended details, see main description in Figure S3. Click here for file

Additional file 22 **Figure S15, Overall forest plot showing the association between rs7248668 and sustained virologic response (SVR)**. Pooled odds ratios were calculated from fixed-effect models with the Mantel-Haenszel method. Superscripts: number of patients with **(a) **favorable genotype (GG) or **(b) **unfavorable genotype (GA+AA) who achieved SVR, with respect to the total number of patients having the favorable or unfavorable genotype, respectively For extended details, see main description in Figure S3. Click here for file

Additional file 23 **Figure S16, Overall forest plot showing the association between rs8103142 and sustained virologic response (SVR)**. Pooled odds ratios were calculated from fixed-effect models with the Mantel-Haenszel method. Superscripts: number of patients with **(a) **favorable genotype (TT) or **(b) **unfavorable genotype (TC+CC) who achieved SVR, with respect to the total number of patients having the favorable or unfavorable genotype, respectively. For extended details, see main description in Figure S3. Click here for file

Additional file 24 **Figure S17, Overall forest plot showing the association between rs8105790 and sustained virologic response (SVR)**. Pooled odds ratios were calculated from fixed-effect models with the Mantel-Haenszel method. Superscripts: number of patients with **(a) **favorable genotype (TT) or **(b) **unfavorable genotype (TC+CC) who achieved SVR, with respect to the total number of patients having the favorable or unfavorable genotype, respectively. For extended details, see main description in Figure S3. Click here for file

Additional file 25 **Figure S18, Overall forest plot showing the association between rs10853728 and sustained virologic response (SVR)**. Superscripts: number of patients with **(a) **favorable genotype (CC) or **(b) **unfavorable genotype (CG+GG) who achieved SVR, with respect to the total number of patients having the favorable or unfavorable genotype, respectively. For extended details, see main description in Figure S3. Click here for file

Additional file 26 **Figure S19, Forest plot showing the associations between *interleukin 28B (IL28B) gene *polymorphisms reported in only one study and sustained virologic response (SVR)**. Superscripts: number of patients with **(a) **favorable genotype (CC) or **(b) **unfavorable genotype (CG+GG) who achieved SVR with respect to the total number of patients having the favorable or unfavorable genotype, respectively. * Single-nucleotide polymorphisms (SNPs) from Smith *et al *article; † SNPs from Chen *et al *article. Click here for file

Additional file 27 **Figure S20, Overall forest plot showing the association between rs12979860 and spontaneous clearance (SC)**. The vertical continuous line indicates no difference in SC for the *interleukin 28B (IL28B) *genotype. Pooled odds ratios were calculated from random-effects models with the DerSimonian-Laird method. **(a) **The number of patients with favorable genotype who achieved SC, with respect to the total number of patients having the favorable genotype. **(b) **The number of patients with unfavorable genotype who achieved SC, with respect to the total number of patients having the unfavorable genotype. For complete details, see main description in Figure S3. Click here for file

Additional file 28 **Figure S21, Forest plot showing the association between rs12979860 and spontaneous clearance (SC) stratified by ethnicity**. The vertical continuous line indicates no difference in SC for the *interleukin 28B (IL28B) *genotype. Pooled odds ratios were calculated from random-effects models with the DerSimonian-Laird method. **(a) **The number of patients with the favorable genotype who achieved SC, with respect to the total number of patients having the favorable genotype. **(b) **The number of patients with unfavorable genotype who achieved SC, with respect to the total number of patients having the unfavorable genotype. For complete details, see main description in Figure S3. Click here for file

Additional file 29 **Figure S22, Forest plot showing the association between rs12979860 and spontaneous clearance (SC) stratified by hepatitis C virus (HCV) genotype**. The vertical continuous line indicates no difference in SC for *interleukin 28B (IL28B) gene *genotype. Pooled odds ratios were calculated from random-effects models with the DerSimonian-Laird method. **(a) **The number of patients with favorable genotype who achieved SC, with respect to the total number of patients having the favorable genotype. **(b) **The number of patients with unfavorable genotype who achieved SC, with respect to the total number of patients having the unfavorable genotype. For complete details, see main description in Figure S3. Click here for file

Additional file 30 **Figure S23, Forest plot showing the association between rs8099917 and spontaneous clearance (SC)**. The vertical continuous line indicates no difference in SC for the *interleukin 28B (IL28B) *genotype. Pooled odds ratios were calculated from fixed-effect models with the Mantel-Haenszel method. **(a) **The number of patients with the favorable genotype who achieved SC, with respect to the total number of patients having the favorable genotype. **(b) **The number of patients with the unfavorable genotype who achieved SC, with respect to the total number of patients having the unfavorable genotype. For complete details, see main description in Figure 3. Click here for file

Additional file 31 **Figure S24, Forest plot showing the association between rs12980275 and spontaneous clearance (SC)**. The vertical continuous line indicates no difference in SC for the *interleukin 28B IL28B) *genotype. Pooled odds ratios were calculated from random-effects models with the DerSimonian-Laird method. **(a) **The number of patients with the favorable genotype who achieved SC, with respect to the total number of patients having the favorable genotype. **(b) **The number of patients with the unfavorable genotype who achieved SC, with respect to the total number of patients having the unfavorable genotype. For complete details, see main description in Figure 3. Click here for file

Additional file 32 **Figure S25, Forest plot showing the associations between *interleukin 28B (IL28B) *gene polymorphisms reported in only one study and spontaneous clearance (SC)**. For extended details, see main description in Figure S3. Click here for file
