# Supplementary material for: Meta-analysis: implications of interleukin-28B polymorphisms in spontaneous and treatment-related clearance for patients with hepatitis C
Source: BMC Med. 2013 Jan 8;11:6. doi: 10.1186/1741-7015-11-6 (PMC3570369; doi:10.1186/1741-7015-11-6)
Supplement: Additional file 1 — Figure S1, Search terms. Relevant studies were identified by a literature search of PubMed without imposing study period restrictions. [file 1741-7015-11-6-S1.PDF]

Additional File 1, Figure S1: Search terms.

Relevant studies were identified by a literature search through PubMed without imposing study period restrictions.

Pubmed:

(("hepatitis c"[MeSH Terms] OR "hepatitis c"[All Fields] OR "hepacivirus"[MeSH Terms] OR "hepacivirus"[All Fields]) OR hcv[All Fields]) AND (IL28b[All Fields] OR IL28[All Fields] OR interleukin-28[All Fields]) AND (SNP[All Fields] OR ("polymorphism, single nucleotide"[MeSH Terms] OR ("polymorphism"[All Fields] AND "single"[All Fields] AND "nucleotide"[All Fields]) OR "single nucleotide polymorphism"[All Fields] OR "snps"[All Fields]) OR ("polymorphism, genetic"[MeSH Terms] OR ("polymorphism"[All Fields] AND "genetic"[All Fields]) OR "genetic polymorphism"[All Fields] OR "polymorphism"[All Fields]) OR ("polymorphism, genetic"[MeSH Terms] OR ("polymorphism"[All Fields] AND "genetic"[All Fields]) OR "genetic polymorphism"[All Fields] OR "polymorphisms"[All Fields]) OR ("genetic variation"[MeSH Terms] OR ("genetic"[All Fields] AND "variation"[All Fields]) OR "genetic variation"[All Fields]) OR ("genotype"[MeSH Terms] OR "genotype"[All Fields]) OR ("alleles"[MeSH Terms] OR "alleles"[All Fields] OR "allele"[All Fields])) AND (clearance[All Fields] OR spontaneous[All Fields] OR ("therapy"[Subheading] OR "therapy"[All Fields] OR "therapeutics"[MeSH Terms] OR "therapeutics"[All Fields]) OR ("therapy"[Subheading] OR "therapy"[All Fields] OR "treatment"[All Fields] OR "therapeutics"[MeSH Terms] OR "therapeutics"[All Fields]) OR ("ribavirin"[MeSH Terms] OR "ribavirin"[All Fields]) OR ("interferons"[MeSH Terms] OR "interferons"[All Fields] OR "interferon"[All Fields]) OR peginterferon[All Fields] OR (pegylated[All Fields] AND ("interferons"[MeSH Terms] OR "interferons"[All Fields] OR "interferon"[All Fields])) OR IFN[All Fields] OR PEG-IFN[All Fields]) NOT (("transplants"[MeSH Terms] OR "transplants"[All Fields] OR "transplant"[All Fields] OR "transplantation"[MeSH Terms] OR "transplantation"[All Fields]) OR ("transplantation, homologous"[MeSH Terms] OR ("transplantation"[All Fields] AND "homologous"[All Fields]) OR "homologous transplantation"[All Fields] OR "allograft"[All Fields]) OR ("liver transplantation"[MeSH Terms] OR ("liver"[All Fields] AND "transplantation"[All Fields]) OR "liver transplantation"[All Fields])) NOT ("review"[Publication Type] OR "review literature as topic"[MeSH Terms] OR "review"[All Fields]).
