# Supplementary material for: Meta-analysis: implications of interleukin-28B polymorphisms in spontaneous and treatment-related clearance for patients with hepatitis C
Source: BMC Med. 2013 Jan 8;11:6. doi: 10.1186/1741-7015-11-6 (PMC3570369; doi:10.1186/1741-7015-11-6)
Supplement: Additional file 2 — Table S1, Summary of characteristics of all studies included for pegylated interferon-α plus ribavirin (PEG-IFN/RBV) treatment outcome and spontaneous clearance. * Data belongs to original sample size, not to the genotyped subpopulation. Abbreviations: A, African; AA, African American; As, Asian; C, Caucasian; H, Hispanic; NA, North African; ND, no data; RCT, randomized controlled trial. [file 1741-7015-11-6-S2.PDF]

Additional File 2, Table S1. Summary of characteristics of all studies included for PEG-IFNα/RBV treatment outcome and spontaneous clearance.

* Data belongs to original sample size, not to the genotyped subpopulation.
Abbreviations: A, African; AA, African American; As, Asian; C, Caucasian; H, Hispanics; NA, North African; ND, no data; RCT, Randomized controlled trial

1a) By SVR

Author (year)	*Type of study*	*IL28B SNPs*	*No. (original sample size)*	*Country (% race)*	*Age (years) Mean or Median*	*Gender (% male)*	*Viral infection*	*HCV GT (%GT 1)*	*HCV viral load* ($x10^5$ IU/ml [a] or log_{10} IU/ml [b])	*Fibrosis score (% F ≥3)*	*SVR (%)*	*HCV Treatment*
Tanaka et al. (2009) [5]	Cohort	rs12980275 rs8099917	314	Japan (100% As)	57.39±9.52	54.46	HCV	1 (100)	*N.D.*	*N.D.* (29.35)	44.59	PEG-IFNα2a PEG-IFNα2b+RBV
Thompson et al. (2010) [25]	Cohort	rs12979860	1587	USA, Spain, Germany, France (73.79% C, 18.9% AA, 7.31%H)	48.34 (43.5-52.3)	60.62	HCV	1 (100)	6.44 (5.7-6.9) [b]	METAVIR (11.73)	40.26	Naive. PEG-IFNα2a PEG-IFNα2b+RBV
Montes-Cano et al. (2010) [26]	Cohort	rs12979860	219 (283)	Spain (100% C)	*N.D.*	58.66 *	HCV	1, 2, 3, 4 (74.2)	5.98 [b]*	Scheuer (28.62)*	51.6	IFNα / IFNα+RBV
Stättermayer et al. (2011) [27]	Cohort	rs12979860, rs8099917	682	Australia (99.56% C)	42.4±11.1	64.81	HCV	1, 2, 3, 4 (54.54)	5.86±0.78 [b] 53.1% <8 [a] 46.9% >8 [a]	Biopsy (33.15)	62.46	Naive PEG-IFNα2a+RBV
Grebely et al. (2010) [28]	Cohort	rs8099917, rs12980275	57 (163)	Australia (91% C)	34.3±9.9*	71.78*	HCV-HCV/HIV	1, 2, 3, 4 (46.9)	*N.D.*	*N.D.*	62.16	Naive PEG-IFNα2a; RBV

Aparicio et al. (2010) [29]	Cohort	rs8099917	160	Spain (100% C)	47.72±0.65	66.87	HCV/HIV	1,3,4 (53.75)	6.77±4.81 [b]	METAVIR (39.02)	41.85	Peg-IFNα+RBV
Dill et al. (2011) [30]	Cohort	rs12979860, rs8099917	109	Switzerland (100% C)	45.21±9.10	63.01	HCV	1,2,3,4 (49.31)	6.56±6.07 [b]	METAVIR (54.79)	45.2	PEG-IFNα2a PEG-IFNα2b+RBV
Kurosaki et al. (2011) [31]	Cohort	rs8099917	496	Japan (100% As)	57.1±9.9	50.40	HCV	1 (100)	82% >6 [a]	METAVIR (24)	38.91	Naive and Prior. Peg-IFN+RBV
Lotrich et al. (2010 [32]	Cohort	rs12979860	133	USA (86.47%C, 13.53%AA)	49.46±10.61	75.44	HCV	1, 2, 3 (73.68)	6.68±6.97 [b]	Ishak (*N.D.*)	46.62	PEG-IFNα2a / PEG-IFNα2b+RBV
Ochi et al. (2011) [33]	Cohort	rs8099917	2104 (2112)	Taiwan (100% As)	56.4±12.05*	57.04*	HCV	1, 2 (74.24)	*N.D.*	Desmet et al.	46.48	Naive and prior. PEG-IFNα2b+RBV
Yu et al. (2011) [34]	Cohort	rs8099917, rs10853728	497	Taiwan (100% As)	52.8±11.1	54.8	HCV	2	5.35±0.92 [b]	knodell and Scheuer (28.4)	89	Naive. PEG-IFNα2a PEG-IFNα2b+ RBV
Nattermann et al. (2011) [111]	Case-control	rs12979860	118 (575)	Germany, Australia, Italy, Spain (100%C)	46.5 (28-73)*	80.89*	HCV/HIV	1 and non-1	5.95 [b]*	*N.D.*	38.98	Naive. PEG-IFNα+RBV
Hsu et al. (2011) [36]	Cohort	rs8099917	91 (145)	Taiwan (100% As)	*N.D.*	*N.D.*	HCV	1, 2 (50.6)	*N.D.*	*N.D.*	81.32	Naive. PEG-IFNα2a+RBV

Chen et al. (2011) [37]	Cohort	rs12980275 rs8105790 rs11881222 rs8103142 rs12979860 rs8099917 rs7248668 rs10853728	728	Taiwan (100% As)	51.2 ±10.4	61	HCV	1 and non-1(58.2)	56.3% <4 [a] 43.7% ≥4 [a]	Ishak (49.5)	76.79	IFNα PEG-IFNα2a PEG-IFNα2b+RBV
Sakamoto et al. (2011) [38]	Cohort	rs8099917	129	Japan (100% As)	64 (20-73)	49.61	HCV	2	6.2 (3.6-7.4) [b]	METAVIR (11.45)	76	PEG-IFNα2a PEG-IFNα2b+ RBV
Moghaddam et al. (2011) [11]	Cohort	rs8099917 rs12979860	281	Scandinavian (100%C)	38 (18-58)	59	HCV	3	36.6% <4 [a] 63.4% >4 [a]	APRI	80.43	PEG-IFNα2b+RBV
Fattovich et al. (2011) [39]	Cohort	rs8099917 rs12979860 rs12980275	280	Italian (100%C)	46 ±11	59	HCV	1, 2, 3 (43.21%)	67% ≥4 [a]	METAVIR (16)	74.68	Naive PEG-IFNα2a PEG-IFNα2b+RBV
Liao et al. (2011) [112]	Cohort	rs12979860	92 (259)	China (100% As)	44.7±15.54	56.52	HCV	1 and non-1 (79.35)	5.86 ±0.95 [b]	Biopsy	63.04	PEG-IFNα+RBV
Sinn et al. (2011) [41]	Cohort	rs8099917	118	Korea (100% As)	56.6±9.2	53	HCV	1, 2 (46.61)	5.90 [b]	Knodell (61)	73.73	Naive (73.73%) and prior (26.27%). PEG-IFNα2a+RBV
Rallon et al. (2011) [42]	Cohort	rs12979860	196	Spain (100% C)	42 (38-45)	75	HCV/HIV	1, 2, 3, 4 (57.14)	6.63 [b]	FibroScan (METAVIR) (30)	53.57	Naive. PEG-IFNα2a PEG-IFNα2b+RBV
Onomoto et al. (2011) [43]	Cohort	rs8099917	72 (87)	Japan (100% As)	59.15 ±9.13*	55.17*	HCV	1	6.26 ±1.46 [b*]	METAVIR (*N.D.*)	43.06	Naive. PEG-IFNα2b+RBV

Lindh et al. (2011)[2][44]	Clinical trial	rs12979860	166 (314)	Sweden (96%C)	42 (20-72)*	62.75*	HCV	2, 3	6.05 [b]*	Ishak (13.06*)	78.91	Naive. PEG-IFNα2a+RBV
Mangia et al. (2011) [45]	RCT	rs12979860	454	Italy (100% C)	50.20±12.36	57.71	HCV	1	6.11 [b]	Scheuer (30.61)	50.66	PEG-IFNα2a PEG-IFNα2b+RBV
De Rueda et al. (2011) [46]	Cohort	rs12979860	423 (428)	Spain (100% C)	≤40=38%; >40=62%	57.94	HCV	1, 2, 3, 4 (1 or 4=88%)	32.94% ≤6 [a]<br>67% >6 [a]	Scheuer (22.87)	56.50	Naive PEG-IFNα2a+RBV
Lindh et al. (2011)[1] [47]	Cohort	rs12979860<br>rs8099917	106 (110)	Sweden (99.1%C)	45.40	60.91	HCV	1	6.42 [b]	Ludvig-Batts (22.58)	59.43	PEG-IFNα2a+RBV
Ladero et al.. (2011)[48]	Cohort	rs12979860<br>rs8099917	110	Spain (100% C)	48 (20-74)	62.72	HCV	1	19.1% <4 [a]<br>80.9% ≥4 [a]	METAVIR (34.7)	46.36	Naive. PEG-IFNα2a PEG-IFNα2b+RBV
Pearlman et al. (2011) [49]	Cohort	rs12979860	90 (101)	USA (52.3%C, 47.7%AA)	55 (27.6-65.5) *	66.34*	HCV	1	6.72 [b]*	METAVIR (26*)	28.89	Prior. Peg-IFNα2b+RBV
Hayashi et al. (2011) [50]	Cohort	rs8099917	299	Japan (100% As)	55.9 ±10.3	52.51	HCV	1	6.24 [b]	METAVIR (15.92)	46.15	PEG-IFNα2b+ RBV
O´Brien et al. (2011)[51]	Cohort	rs12979860	646	USA (100%C)	49 (45-53)	75.39	HCV	1	6.5 (6.2-6.8) [b]	Ishak (54.80)	14.24	Prior. PEG-IFNα2a+RBV
Smith et al. (2011)[52]	Cohort	rs8105790<br>rs11881222<br>rs7248668<br>rs10853728	817 (905)	Germany (100%C)	*N.D.*	*N.D.*	HCV	1	*N.D.*	*N.D.*	45.66	Peg-IFN+RBV
Halfon et al. (2011)[53]	Cohort	rs8099917<br>rs12979860	198	France (100% C)	47±12	70.70	HCV	1, 2, 3 (78.78)	45% <4[a]<br>55% ≥4[a]	METAVIR (37.37)	54.54	Peg-IFN+RBV
Lyoo et al. (2011) [54]	Cohort	rs8099917<br>rs12979860	65	Korea (100% As)	52.4± 9.7	56.92	HCV	1	7.5 ±5.0 [a]	*N.D.*	64.61	PEG-IFNα2a+RBV

De Nicola et al. (2012) [55]	Cohort	rs12979860	103 (112)	Italy (38.08% C, 61.92%NA)	45	87.50*	HCV	4	65%<6 [a] 42.86%>6[a]	METAVIR (*N.D.*)	49.54	Naive. PEG-IFNα2a PEG-IFNα2b+RBV
Asselah et al. (2011) [56]	Cohort	rs12979860	82 (164)	France, Egypt (34.2%C, 51.2%NA, 14.6% others)	44	76.80	HCV	4	*N.D.*	METAVIR (37.18)	52.44	PEG-IFNα2a PEG-IFNα2b+RBV
Venegas et al. (2011) [9]	Cohort	rs12979860 rs12980275 rs8099917	99	Chile (69%H,31% Native)	*N.D.*	*N.D.*	HCV	1	*N.D.*	*N.D.*	50.5	Naive. PEG-IFNα2a+RBV
Patel et al. (2011) [10]	Cohort	rs12979860	41 (96)	USA (78.05%C, 21.95% others)	48.10	60.97	HCV	1	*N.D.*	METAVIR (*N.D.*)	63.41	PEG-IFNα+RBV
Chevaliez et al. (2011) [57]	Clinical trial	rs12979860	83 (104)	France (Mix)	50 (34-68)	68.67	HCV	1	6.5±0.6 [b]	Fibrotest and/or Fibroscan (*N.D.*)	6.02	PEG-IFNα2a+RBV
Huang et al. (2012)[58]	Cohort	rs8099917	226	Taiwan (100% As)	51.90	73.08	HCV	1	5.4[b]	*N.D.*	57.96	Naïve. PEG-IFNα2a or PEG-IFNα2b+RBV
Howell et al. (2012)[59]	Cohort	rs12979860	361	USA (52.1%C, 47.9%AA)	48.06	57.34	HCV	1	6.45 (5.6-6.8) [b]	Ishak(*N.D.)*	40.44	Naïve PEG-IFNα2a+RBV
Miyaaki et al. (2011)[60]	Cohort	rs8099917	67	Japan (100% As)	56.8 ± 9.3	55.22	HCV	1	6.36 ± 6.18 [b]	New Inuyama classification *(N.D.)*	43.28	Naïve PEG-IFNα+RBV
Lange et al. (2012)[61]	Cohort	rs8099917	501 (876)	Swiss (93%C)	45 (19-75)	65	HCV-HCV/HIV	1, 3 (56.49)	5.96 (1-8) [b]	METAVIR (68)	58.30	PEG-IFNα2a or PEG-IFNα2b+RBV

Cavalcante et al. (2012)[62]	Case-control	rs12979860 rs8099917	222	Brazil (41.1%C, 58.9 A)	52.30	42.47	HCV	1, 2, 3 (74.8)	5.79 (5.1-6.1) [b]	METAVIR (53.7)	39.64	PEG-IFNα2a or PEG-IFNα2b+RBV
Payer et al. (2012)[63]	Cohort	rs12979860	69 (72)	Austria (100%C)	38.4 (± 9.3)*	72*	HCV/HIV	1, 2, 3, 4 (58.3)	*N.D.*	METAVIR (35)	62.30	Naïve. PEG-IFNα2a+RBV
Miyamura et al. (2011)[64]	Cohort	rs8099917	79 (202)	Japan (100%As)	55.4 ± 11.7	50	HCV	1, 2, unknown (82.2)	*N.D.*	*Biopsy (31.6)*	36.70	PEG-IFNα2a+RBV
Di Marco et al. (2012)[65]	Cohort	rs12979860 rs8099917	114 (301)	Italy (100%C)	21.37	32.31	HCV	1, 2, 3, 4 (unknown)	*N.D.*	Scheuer (*N.D.)*	40.35	Naïve. IFNα monotherapy
Takita (2011)[66]	Cohort	rs8099917	122	Japan (100%As)	61 (20-78)	79.40	HCV	1 (100)	*N.D.*	*N.D.* (14.75*)*	51.60	PEG-IFNα2a or PEG-IFNα2b+RBV
Fischer et al. (2012)[67]	Case-control	rs12979860 rs8099917 rs12980275 rs8103142	1319	Australia, England, Germany, Italy (100%C)	47.7 ± 11	56.33	HCV	1 (100)	29.5% < 5.6 ≥70.5% [b]	*Biopsy (N.D.)*	48.22	PEG-IFNα+RBV
Liu et al. (2012)[68]	Cohort	rs8099917	662 (791)	Taiwan (100%As)	54.5 ± 10.5	57.10	HCV	1 (100)	5.85 ± 0.7 [b]	METAVIR *(N.D.)*	62.83	Naïve. PEG-IFNα2a+RBV
Younossi et al. (2012)[69]	Cohort	rs12979860	56	USA (60%C, 20% others)	48.52 ± 5.96	62.50	HCV	Mix (76.8)	6.7 ± 6.8 [b]	*N.D.*	41.07	Naive and prior. PEG-IFNα2a or PEG-IFNα2b+RBV
Vidal-Castiñeira et al. (2012)[70]	Cohort	rs12979860	407	Spain (100%C)	46.45	70.50	HCV	1, 2, 3, 4 (76.90)	*22.36% <5.6* [b]	FibroScan *(N.D.)*	51.59	Naive. PEG-IFNα2a or PEG-IFNα2b+RBV
Sporea et al. (2011)[71]	Cohort	rs12979860	106 (107)	Romania (100%C)	53.4±9.6*	41.10	HCV	1 (99.7)	6.45 [b]	METAVIR (47.06)	50.94	PEG-IFNα2a or PEG-IFNα2b+RBV

Miyase et al. (2012)[72]	Clinical trial	rs8099917	201	Japan (100%As)	59.05±9.94	39.30	HCV	1 (100)	6.25±0.65 [b]	METAVIR *(N.D.)*	58.21	PEG-IFNα2a or PEG-IFNα2b+RBV
Li et al. (2012)[73]	Cohort	rs8099917	56	China (100%As)	37.7±10.9	51.78	HCV	1 (100)	*N.D.*	*N.D.*	60.71	Peg-IFN+RBV
Inokuchi et al. (2012)[74]	Cohort	rs8099917	99 (102)	Japan (100%As)	54.1±11.4*	51.96*	HCV	1, 2 (75.5%)	5.9±0.82 [b]	Desmet et al. (16.3)	59.59	Peg-IFN+RBV
Kobayashi et al. (2012)[75]	Cohort	rs12979860	995 (1516)	Japan (100%As)	50 (13-81)*	58.40*	HCV	1, 2 (68.9%)	*N.D.*	*N.D.*	62.33	IFN monotherapy or PEG-IFNα2b+RBV
Kim et al. (2012)[76]	Cohort	rs8099917	75	Japan (100%As)	60 (30-74)	57.33	HCV	1 (100)	6.2 (5.0-7.1) [b]	*N.D.*	49.33	PEG-IFNα2b+RBV
Petta et al. (2012)[77]	Cohort	rs12979860	117	Italy (100%C)	51.9±11.3	50.40	HCV	1 (100)	5.95±6.07 [b]	Scheuer (25.64)	49.57	Naïve. PEG-IFNα2a+RBV
Tajir et al. (2012)[78]	Cohort	rs12979860	60	Morocco (100%NA)	*N.D.*	*N.D.*	HCV	1 (100)	*N.D.*	*N.D.*	53.33	Peg-IFN+RBV
Valenti et al. (2012)[79]	Cohort	rs12979860	602	Austria, Italy (100%C)	51±12	61	HCV	1, 2, 3, 4 (61)	5.9±0.8 [b]	Ishak and METAVIR *(N.D.)*	58.30	PEG-IFNα2a or PEG-IFNα2b+RBV
Saito et al. (2012)[80]	Cohort	rs8099917	226 (696)	Japan (100%As)	57.33±0.84	47.34	HCV	1 (100)	6.2±0.1 [b]	*N.D.*	*41.15*	PEG-IFNα2a or PEG-IFNα2b+RBV
Karchava et al. (2012)[81]	Cohort	rs12979860	143 (156)	USA (100%C)	40 (32-46)	82.50	HCV	1, 2, 3 (34.9)	*N.D.*	FibroScan *(N.D.)*	62.94	Naive. PEG-IFNα2a or PEG-IFNα2b+RBV
Ogawa et al. (2012)[82]	Cohort	rs8099917	328	Japan (100%As)	59 (54-65)	44.80	HCV	1 (100)	6.2 (5.9-6.6) [b]	Bedossa et al. (28.49)	35.67	Naive. PEG-IFNα2a or PEG-IFNα2b+RBV

Tolmane et al. (2012)[83]	Cohort	rs12979860	123 (142)	Latvia (100%C)	37(18-68)*	59*	HCV	1, 2, 3 (46)	6.37 [b]	Knodell (16.67)	68.29	PEG-IFNα2a or PEG-IFNα2b+RBV
Christensen et al. (2012)[84]	Cohort	rs12979860	44 (46)	Denmark(*N.D.)*	45.39 (39.43-50.35)*	76.09*	HCV	1, 2, 3, 4 (30.43)	5.7 [b]	*N.D.*	63.64	PEG-IFNα2a+RBV
Mira et al. (2012)[85]	Cohort	rs12979860	84 (124)	Spain (*N.D.)*	42 (39-46)*	82*	HCV/HIV	4 (0)	5.6 (5.1-6.0) [b]	Scheuer (N.D.)	28.57	Naive. PEG-IFNα2a or PEG-IFNα2b+RBV
Amanzada et al. (2012)[86]	Cohort	rs12979860	305	Germany, USA (97%C)	52±13	55	HCV	1 (100)	*N.D.*	Desmet et al. (13.47)	43.93	Naïve and prior. PEG-IFNα2a or PEG-IFNα2b+RBV
Guo et al. (2012)[87]	Cohort	rs8099917	236	China (100%As)	44.10±11.54	63.56	HCV	1 (100)	6.14±1.07 [b]	Desmet et al. (40.62)	46.03	PEG-IFNα2a+RBV

1b) By SC

Author (year)	*Type of study*	*IL28B SNPs*	*No. (original sample size)*	*Country (% race)*	*Age (years) Mean or Median*	*Gender (% male)*	*Viral infection*	*HCV genotype (% genotype-1)*	*SC (%)*
Montes-Cano et al. (2010)[26]	Cohort	rs12979860	352	Spain (100% C)	58.66	*N.D.*	HCV	1,2,3,4 (74.2)	19.6
Grebely et al. (2010)[28]	Cohort	rs8099917 rs12980275	75-79 (163)	Australia (91% C) *	34.3±9.9*	71.78*	HCV (69%); HCV/HIV (31%)*	1,2,3,4	23
Dring et al. (2011)[88]	Cohort	rs12979860	536 (543)	Ireland (100% C)	27.4±5.5	0	HCV	1 (100)	45.15
Knapp et al. (2011)[89]	Cohort	rs12979860	323 (397)	United Kingdom (94.12% C)	42.52	60.93	HCV	*N.D.*	27.55
Renda et al. (2011)[90]	Cohort	rs8103142	42	Italy (100% C)	44.68±12.09	38.10	HCV	1,2 (90.5)	47.62
Nattermann et al. (2011) [91]	Cohort	rs12979860	396	Germany (100% C)	24.7±4	0	HCV	1 (100)	30.05
Suppiah et al. (2011) [92]	Cohort	rs8099917	1091	Australia, Germany, Italy, UK, Switzerland (100% C)	*N.D.*	52.60	HCV	1 (100)	19.98
Mangia et al. (2012) [93]	Cohort	rs12979860	117	Italy (100% C)	35.2 ± 5.9	50	HCV	1(56)	58.20
Di Marco et al. (2012) [65]	Cohort	rs12979860 rs8099917	245 (301)	Italy (100% C)	N.D.	N.D.	*N.D.*	1 (78.9)	40.00
Rao et al. (2012) [94]	Cohort	rs12979860 rs8099917 rs11881222 rs10853728 rs12980275 rs4803219 rs4803223 rs8105790	376	China (100% As)	53.2±8.0	76.52	HCV	1, 2 (>83.8)	21.28
