# Supplementary material for: Meta-analysis: implications of interleukin-28B polymorphisms in spontaneous and treatment-related clearance for patients with hepatitis C
Source: BMC Med. 2013 Jan 8;11:6. doi: 10.1186/1741-7015-11-6 (PMC3570369; doi:10.1186/1741-7015-11-6)
Supplement: Additional file 3 — Table S2, Quality appraisal for meta-analysis of sustained virologic response (SVR). 1) Source population well described? 2) Population well described and appropriate? 3) Did participants represent those eligible? 4) Was information on previous hepatitis C virus (HCV) treatment information reported? 5) Inclusion/exclusion criteria reported? 6) Type of treatment well described? 7) Outcome measures well described and without incongruencies? 8) Outcome measurement complete? (That is, all genotype counts reported?) 9) Assessment of Hardy-Weinberg equilibrium? 10) Description of what genetic model was assumed? 11) Consideration of genotyping errors and confirmation of results? 12) Information of linkage disequilibrium? 13) Information on haplotypes? 14) Responder and non-responder groups comparison at baseline? 15) Raw data given or calculable? 16) Study sufficiently powered? 17) Statistical methods appropriate? 18) Study results internally valid (that is, unbiased)? (Summary of items 5 to 8 and 14 to 17)? 19) Genetic study reliable? (Summary of items 9 to 13). 20) Results generalizable to the source population (that is, external validity)? (Summary of items 1 to 4). 21) Overall study quality? Abbreviations: NA, not applicable; NR, not reported. [file 1741-7015-11-6-S3.PDF]

Additional File 3, Table S2. Quality appraisal for SVR meta-analysis.

Items description: 1. Source population well described? 2. Population well described and appropriate? 3.Participants represent elegibles? 4. Was prior HCV treatment information reported? 5. Inclusion/Exclusion criteria reported. 6. Type of treatment well described. 7. Outcome measures well described and without incongruences? 8. Outcome measurement complete? (All genotype counts reported). 9. Assessment of Hardy-Weinberg equilibrium. 10. Description of what genetic model was assumed. 11. Consideration of genotyping errors/confirmation of results. 12. Information of linkage disequilibrium. 13. Information on haplotypes. 14. Responder and non-responder groups comparison at baseline? 15. Raw data given or calculable? 16. Study sufficiently powered? 17. Statistical methods appropriate? 18. Study results internally valid (i.e. unbiased)? (numbers 5-8 and 14-17). 19. Genetic study reliable? (numbers 9-13). 20. Results generalizable to the source population (i.e. external validity)? (numbers 1-4). 21. Overall study quality. Abbreviations: NA, not applicable; NR: not reported.

Items	Tanaka et al (2009)	Thompson et al (2010)	Montes-Cano et al (2010)	Stättermayer et al (2011)	Grebely et al (2010)	Aparicio et al (2010)	Dill et al (2011)	Kurosaki et al (2011)	Lotrich et al (2010)	Ochi et al (2010)	Yu et al (2011)	Nattermann et al (2011)	Hsu et al (2011)	Chen et al (2011)	Sakamoto et al (2011)	Moghaddam et al (2011)	Fattovich et al (2011)
**1**	0	+1	+1	-1	+1	-1	+1	0	+1	+1	-1	-1	+1	+1	0	+1	0
**2**	0	+1	0	+1	0	+1	+1	+1	+1	0	+1	0	-1	0	+1	0	+1
**3**	+1	0	0	+1	-1	+1	-1	+1	+1	0	0	0	-1	+1	+1	+1	+1
**4**	-1	+1	-1	+1	+1	-1	-1	-1	-1	-1	+1	-1	+1	-1	-1	+1	+1
**5**	+1	0	1	0	+1	+1	0	+1	0	+1	+1	0	+1	0	+1	+1	+1
**6**	+1	+1	0	+1	+1	0	0	+1	+1	+1	+1	0	+1	+1	+1	+1	+1
**7**	+1	+1	0	+1	+1	+1	+1	+1	+1	-1	+1	0	+1	+1	+1	+1	0
**8**	+1	+1	0	+1	+1	+1	+1	+1	+1	+1	0	+1	+1	+1	0	+1	+1
**9**	+1	-1	1	0	+1	+1	-1	-1	-1	+1	NR	+1	-1	+1	-1	+1	-1
**10**	+1	-1	0	-1	-1	-1	0	-1	-1	+1	-1	-1	-1	-1	-1	-1	-1
**11**	+1	-1	-1	-1	-1	-1	-1	-1	-1	-1	-1	-1	-1	-1	-1	+1	+1
**12**	+1	NA	NA	-1	-1	NA	-1	NA	NA	+1	+1	NA	NA	+1	NA	-1	-1
**13**	+1	NA	NA	-1	-1	NA	-1	NA	NA	0	-1	NA	NA	+1	NA	-1	-1
**14**	+1	-1	-1	0	-1	+1	0	-1	+1	-1	+1	-1	-1	+1	+1	-1	+1
**15**	+1	+1	1	+1	-1	-1	+1	-1	-1	+1	+1	+1	+1	-1	+1	+1	+1
**16**	+1	+1	1	+1	-1	0	-1	+1	0	+1	+1	0	-1	+1	0	+1	+1
**17**	+1	+1	0	+1	+1	+1	+1	+1	0	+1	+1	+1	+1	+1	+1	+1	+1
**18**	+1	+1	0	+1	+1	+1	0	+1	0	+1	+1	0	+1	+1	+1	+1	+1
**19**	+1	-1	0	-1	-1	-1	-1	-1	-1	+1	-1	0	-1	+1	-1	0	-1
**20**	0	+1	0	+1	0	0	0	0	+1	0	0	0	0	0	0	+1	+1
**21**	+1	+1	0	+1	0	0	0	0	0	+1	0	0	0	+1	0	+1	+1

Items	Liao et al (2011)	Sinn et al (2011)	Rallón et al (2011)	Onomoto et al (2011)	Lindh et al (2011)	Mangia et al (2011)	De Rueda et al (2011)	Lindh et al (2011)	Ladero et al (2011)	Pearlman et al (2011)	Hayashi et al (2011)	O´Brien et al (2011)	Smith et al (2011)	Halfon et al (2011)	Lyoo et al (2011)	De Nicola et al (2012)	Asselah et al (2011)
**1**	+1	+1	+1	+1	-1	0	+1	-1	+1	+1	+1	+1	0	-1	+1	+1	+1
**2**	0	+1	+1	0	+1	+1	+1	+1	+1	+1	+1	+1	-1	+1	0	+1	0
**3**	-1	+1	+1	0	-1	+1	0	0	+1	0	+1	+1	0	+1	+1	0	-1
**4**	-1	+1	+1	+1	+1	-1	+1	-1	+1	+1	-1	+1	-1	-1	-1	+1	-1
**5**	+1	+1	+1	+1	0	+1	+1	-1	+1	+1	+1	+1	+1	0	-1	+1	+1
**6**	0	+1	+1	+1	+1	+1	+1	+1	+1	+1	+1	+1	0	0	+1	+1	+1
**7**	+1	+1	+1	+1	+1	+1	+1	+1	0	+1	+1	+1	+1	+1	+1	+1	+1
**8**	+1	0	0	+1	+1	+1	0	+1	+1	0	+1	+1	+1	0	+1	+1	+1
**9**	+1	-1	-1	-1	-1	+1	-1	-1	+1	-1	-1	-1	+1	-1	-1	-1	+1
**10**	-1	-1	-1	-1	-1	-1	-1	+1	-1	-1	-1	-1	+1	0	-1	-1	0
**11**	-1	+1	-1	-1	-1	-1	-1	-1	+1	-1	-1	-1	+1	-1	-1	-1	-1
**12**	NA	0	NA	NA	NA	NA	NA	+1	+1	NA	NA	NA	+1	-1	-1	NA	NA
**13**	NA	-1	NA	NA	NA	NA	NA	+1	+1	NA	NA	NA	+1	-1	-1	NA	NA
**14**	0	+1	-1	+1	-1	-1	+1	-1	0	-1	+1	-1	+1	+1	-1	+1	-1
**15**	+1	+1	-1	+1	0	+1	+1	0	+1	+1	+1	-1	+1	0	-1	+1	-1
**16**	-1	0	0	-1	0	+1	+1	0	0	-1	+1	+1	+1	0	-1	0	-1
**17**	+1	0	+1	NA	+1	+1	+1	+1	+1	-1	+1	+1	0	0	-1	+1	-1
**18**	+1	+1	0	+1	0	+1	+1	0	+1	0	+1	+1	+1	0	-1	+1	0
**19**	0	-1	-1	-1	-1	-1	-1	+1	+1	-1	-1	-1	+1	-1	-1	-1	0
**20**	0	+1	+1	0	0	0	+1	0	+1	+1	+1	+1	0	0	0	+1	0
**21**	0	+1	0	0	0	0	+1	0	+1	0	+1	+1	+1	0	-1	+1	0

Items	Venegas et al (2011)	Patel et al (2011)	Chevaliez et al (2011)	Huang et al (2012)	Howell et al (2012)	Miyaaki et al (2012)	Lange et al (2012)	Cavalcante et al (2012)	Payer et al (2012)	Miyamura et al (2011)	Di Marco et al (2012)	Takita et al (2011)	Fischer et al (2012)	Liu et al (2012)	Younossi et al (2012)	Vidal-Castiñeira et al (2012)	Sporea et al (2011)	Miyase et al (2012)	Li et al (2012)	Inokuchi et al (2012)	Kobayashi et al (2012)	Kim et al (2012)	Petta et al (2012)	Tajir et al (2012)	Valenti et al (2012)	Saito et al (2012)	Karchava et al (2012)	Ogawa et al (2012)	Tolmane et al (2012)	Christensen et al (2012)	Mira et al (2012)	Amanzada et al (2012)	Guo et al (2012)
**1**	+1	0	+1	0	0	+1	0	-1	+1	+1	0	+1	+1	-1	-1	+1	0	+1	-1	+1	+1	+1	+1	-1	+1	+1	+1	+1	+1	-1	-1	-1	+1
**2**	-1	0	+1	+1	+1	+1	+1	+1	0	+1	0	0	0	0	0	0	0	0	-1	+1	0	0	+1	-1	+1	0	+1	+1	+1	0	0	0	+1
**3**	+1	-1	0	+1	+1	+1	-1	+1	0	-1	-1	+1	+1	0	+1	+1	0	+1	+1	0	-1	+1	+1	+1	+1	-1	0	+1	0	0	-1	+1	+1
**4**	+1	-1	+1	+1	+1	+1	-1	-1	+1	-1	+1	-1	-1	+1	+1	+1	-1	+1	NA	-1	+1	-1	+1	-1	+1	-1	+1	+1	-1	-1	+1	+1	-1
**5**	+1	0	+1	+1	-1	0	0	+1	0	0	0	-1	-1	-1	-1	0	-1	+1	NA	-1	+1	0	+1	-1	+1	+1	+1	+1	-1	+1	0	+1	+1
**6**	0	0	+1	+1	+1	+1	0	0	+1	+1	+1	+1	0	+1	0	+1	+1	+1	-1	-1	+1	+1	0	-1	0	+1	+1	+1	0	+1	+1	+1	+1
**7**	0	+1	+1	+1	0	+1	0	+1	-1	+1	+1	0	+1	+1	+1	+1	0	+1	+1	0	+1	+1	+1	+1	0	+1	+1	+1	-1	+1	+1	+1	+1
**8**	+1	0	+1	0	+1	0	0	+1	0	0	0	0	+1	0	0	+1	+1	0	+1	0	+1	0	0	+1	0	0	0	+1	0	+1	+1	+1	+1
**9**	-1	-1	-1	-1	-1	-1	NR	+1	-1	-1	NR	-1	0	+1	-1	-1	-1	-1	-1	-1	-1	-1	-1	NR	-1	-1	-1	-1	-1	-1	-1	+1	-1
**10**	0	-1	-1	-1	0	-1	-1	-1	-1	-1	+1	-1	+1	-1	-1	-1	-1	-1	0	-1	0	-1	-1	-1	-1	-1	-1	-1	-1	-1	-1	-1	0
**11**	-1	-1	-1	-1	-1	0	-1	-1	-1	-1	+1	-1	+1	-1	-1	+1	-1	-1	+1	-1	-1	-1	-1	-1	-1	-1	-1	-1	-1	-1	-1	-1	-1
**12**	-1	NA	NA	NA	NA	NA	NA	+1	NA	NA	-1	NA	+1	NA	NA	NA	NA	NA	0	NA	+1	NA	-1	NA	NA	NA	NA	NR	NA	NA	NA	-1	NA
**13**	-1	NA	NA	NA	NA	NA	NA	-1	NA	NA	-1	NA	+1	NA	NA	NA	NA	NA	-1	NA	-1	NA	-1	NA	NA	NA	NA	NR	NA	NA	NA	-1	NA
**14**	0	+1	+1	+1	-1	0	0	+1	+1	+1	+1	+1	+1	+1	NA	+1	-1	-1	NA	+1	-1	+1	+1	-1	-1	+1	-1	+1	-1	0	-1	+1	+1
**15**	-1	+1	+1	+1	-1	+1	+1	-1	+1	+1	+1	-1	-1	+1	+1	+1	+1	-1	+1	+1	-1	+1	+1	+1	0	+1	+1	+1	0	+1	+1	+1	+1
**16**	-1	-1	-1	+1	+1	-1	+1	+1	-1	-1	0	0	+1	+1	-1	+1	0	+1	-1	-1	+1	-1	0	-1	+1	+1	0	+1	0	-1	-1	+1	+1
**17**	-1	NA	0	+1	+1	0	+1	-1	NA	-1	+1	0	+1	+1	NA	+1	-1	+1	NA	+1	-1	+1	+1	-1	NA	+1	-1	+1	-1	+1	+1	+1	+1
**18**	0	0	+1	+1	0	0	0	+1	0	0	+1	0	+1	+1	0	+1	0	+1	+1	0	+1	+1	+1	-1	0	+1	0	+1	0	+1	+1	+1	+1
**19**	-1	-1	-1	-1	-1	-1	-1	0	-1	-1	0	-1	+1	-1	-1	-1	-1	-1	0	-1	-1	-1	-1	-1	-1	-1	-1	-1	-1	-1	-1	-1	-1
**20**	+1	0	+1	+1	+1	+1	0	0	0	0	0	0	0	0	0	+1	0	+1	0	0	0	0	+1	-1	+1	0	+1	+1	0	0	0	0	+1
**21**	0	0	+1	+1	0	0	0	0	0	0	0	0	+1	0	0	+1	0	+1	0	0	0	0	+1	-1	0	0	0	+1	0	0	0	0	+1
