# Supplementary material for: Meta-analysis: implications of interleukin-28B polymorphisms in spontaneous and treatment-related clearance for patients with hepatitis C
Source: BMC Med. 2013 Jan 8;11:6. doi: 10.1186/1741-7015-11-6 (PMC3570369; doi:10.1186/1741-7015-11-6)
Supplement: Additional file 4 — Table S3, Quality appraisal for SC meta-analysis. 1) Source population well described? 2) Population well described and appropriate? 3) Did participants represent those eligible? 4)? Was information on previous hepatitis C virus (HCV) reported? 5) Inclusion/exclusion criteria reported? 6) Type of treatment well described? 7) Outcome measures well described and without incongruencies? 8) Outcome measurement complete? (That is, all genotype counts reported?) 9) Assessment of Hardy-Weinberg equilibrium? 1) Description of what genetic model was assumed? 11) Consideration of genotyping errors and confirmation of results? 12) Information on linkage disequilibrium? 13) Information on haplotypes? 14) Spontaneous responder and non-responder groups comparison at baseline? 15? Raw data given or calculable? 16) Study sufficiently powered? 17) Statistical methods appropriate? 18) Study results internally valid (that is, unbiased)? (summary of items 5 to 8 and 14 to 17.) 19) Genetic study reliable? (summary of items 9 to 13.) 20) Results generalizable to the source population (that is, external validity)? (summary of items 1 to 4.) 21) Overall study quality? Abbreviations: NA, not applicable; NR, not reported. [file 1741-7015-11-6-S4.PDF]

Additional File 4, Table S3. Quality appraisal for SC meta-analysis.

Items description: 1. Source population well described? 2. Population well described and appropriate? 3.Participants represent elegibles? 4. Was prior HCV treatment information reported? 5. Inclusion/Exclusion criteria reported. 6. Type of treatment well described. 7. Outcome measures well described and without incongruences? 8. Outcome measurement complete? (All genotype counts reported). 9. Assessment of Hardy-Weinberg equilibrium. 10. Description of what genetic model was assumed. 11. Consideration of genotyping errors/confirmation of results. 12. Information of linkage disequilibrium. 13. Information on haplotypes. 14. Spontaneous responder and non-responder groups comparison at baseline? 15. Raw data given or calculable? 16. Study sufficiently powered? 17. Statistical methods appropriate? 18. Study results internally valid (i.e. unbiased)? (numbers 5-8 and 14-17). 19. Genetic study reliable? (numbers 9-13). 20. Results generalizable to the source population (i.e. external validity)? (numbers 1-4). 21. Overall study quality. Abbreviations: NA, not applicable; NR: not reported.

**Items**	Montes-Cano et al (2010)	Grebely et al (2010)	Dring et al (2011)	Knapp et al (2011)	Renda et al (2011)	Nattermann et al (2011)	Suppiah et al (2011)	Mangia et al (2012)	Di Marco et al (2012)	Rao et al (2012)
**1**	1	1	-1	1	-1	0	0	+1	0	0
**2**	0	1	1	1	0	+1	0	0	-1	+1
**3**	1	-1	0	0	1	+1	+1	+1	+1	+1
**4**	NA	NA	NA	NA	NA	NA	NA	NA	NA	NA
**5**	1	1	1	1	0	0	+1	+1	0	0
**6**	NA	NA	NA	NA	NA	NA	NA	NA	NA	NA
**7**	0	1	1	1	1	+1	+1	+1	+1	0
**8**	0	1	1	1	1	+1	+1	+1	0	0
**9**	1	1	1	-1	-1	+1	-1	+1	NR	0
**10**	0	-1	0	-1	-1	-1	0	0	+1	-1
**11**	-1	-1	0	-1	-1	-1	-1	-1	+1	-1
**12**	NA	-1	NA	NA	-1	NA	-1	NA	-1	+1
**13**	NA	-1	NA	NA	-1	NA	+1	NA	-1	+1
**14**	-1	-1	-1	0	-1	-1	0	+1	-1	-1
**15**	-1	-1	1	1	1	+1	+1	+1	-1	+1
**16**	1	-1	1	1	-1	+1	+1	0	+1	+1
**17**	0	1	0	0	-1	+1	+1	+1	+1	-1
**18**	0	+1	+1	+1	0	+1	+1	+1	0	0
**19**	0	-1	0	-1	-1	0	-1	0	0	0
**20**	+1	+1	0	+1	0	+1	0	+1	0	+1
**21**	0	+1	0	+1	0	+1	0	+1	0	0
