# Supplementary material for: Meta-analysis: implications of interleukin-28B polymorphisms in spontaneous and treatment-related clearance for patients with hepatitis C
Source: BMC Med. 2013 Jan 8;11:6. doi: 10.1186/1741-7015-11-6 (PMC3570369; doi:10.1186/1741-7015-11-6)

Additional File 5, Figure S2. The location of studied SNPs in the genome.

* SNPs most studied in recent years

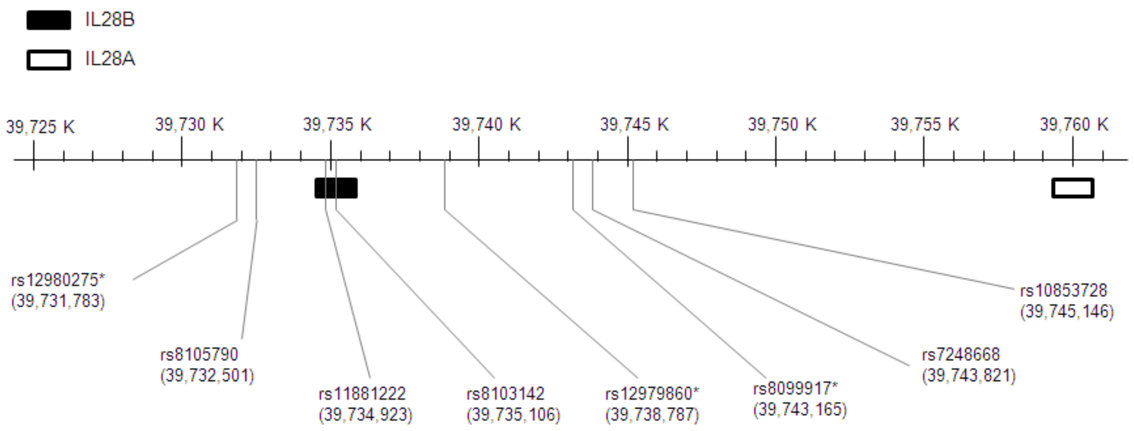

Supplement: Additional file 5 — Figure S2, The location of the studied single-nucleotide polymorphisms (SNPs) in the genome. * SNPs most studied in recent years. [file 1741-7015-11-6-S5.PDF]
