# Supplementary material for: Meta-analysis: implications of interleukin-28B polymorphisms in spontaneous and treatment-related clearance for patients with hepatitis C
Source: BMC Med. 2013 Jan 8;11:6. doi: 10.1186/1741-7015-11-6 (PMC3570369; doi:10.1186/1741-7015-11-6)
Supplement: Additional file 6 — Table S4, Identification of all subgroups available for each study. [file 1741-7015-11-6-S6.PDF]

Additional File 6, Table S4. Identification of all subgrups available on each study. NA: not applicable

	Subgrups					
Study	**a**	**b**	**c**	**d**	**e**	**f**
**Thompson et al. (2010)**	Caucasian	African-American	Hispanics	NA	NA	NA
**Montes-Cano et al. (2010)**	IFNα monotherapy group	IFNα+RBV combined therapy group	NA	NA	NA	NA
**Stättermayer et al. (2011)**	Genotype 1	Genotype 2,3	Genotype 4	NA	NA	NA
**Grebely et al. (2010)**	Genotype 1,4	Genotype 2,3	NA	NA	NA	NA
**Aparicio et al. (2010)**	Genotype 1	Genotype 3	Genotype 4	NA	NA	NA
**Dill et al. (2011)**	Genotype 1	Genotype 2	Genotype 3	Genotype 4	NA	NA
**Ochi et al. (2011)**	Set1 (IFN+RBV, GT1)	Set2 (IFN+RBV, GT1)	Set3 (IFN, GT1)	Set4 (IFN, GT2)	Set5 (IFN+RBV, GT1)	Set6 (IFN+RBV, GT2)
**Fattovich et al. (2011)**	Genotype 1	Genotype 2	Genotype 3	NA	NA	NA
**Sinn et al. (2011)**	Genotype 1	Genotype 2	NA	NA	NA	NA
**Rallon et al. (2011)**	Genotype 1,4	Genotype 2,3	NA	NA	NA	NA
**Halfon et al. (2011)**	Genotype 1	Genotype 2,3	NA	NA	NA	NA
**De Nicola et al. (2012)**	Egyptian patients	Italian patients	NA	NA	NA	NA
**Howell et al. (2011)**	African-Americans	Caucasians	NA	NA	NA	NA
**Lange et al. (2012)**	Genotype 1	Genotype 3	NA	NA	NA	NA
**Cavalcante et al. (2012)**	Mix race, Genotype 1	Mix race, Genotype 2,3	Caucasian, Genotype 1,2,3	African, Genotype 1,2,3	NA	NA
**Payer et al. (2012)**	Genotype 1, 4	Genotype 2,3	NA	NA	NA	NA
**Inokuchi et al. (2012)**	Genotype 1	Genotype 2	NA	NA	NA	NA
**Kobayashi et al. (2012)**	Peg-IFNα2b+RBV and genotype 1	Peg-IFNα2b+RBV and genotype 2	IFN monotherapy and genotype 1	IFN monotherapy and genotype 2	NA	NA
**Karchava et al. (2012)**	Genotype 1	Genotype 2	NA	NA	NA	NA
**Christensen et al. (2012)**	Genotype 1,4	Genotype 2,3	NA	NA	NA	NA
