# Supplementary material for: Meta-analysis: implications of interleukin-28B polymorphisms in spontaneous and treatment-related clearance for patients with hepatitis C
Source: BMC Med. 2013 Jan 8;11:6. doi: 10.1186/1741-7015-11-6 (PMC3570369; doi:10.1186/1741-7015-11-6)
Supplement: Additional file 7 — Table S5, Egger test result bias for rs12979860 and rs8099917 for sustained virologic response (SVR). No., number of studies; Coef., asymmetry regression coefficient; Std. Err., standard error; t, statistic; P > |t|, significance; and 95% CI, confidence interval. Coef. corresponds to the intercept value in the regression equation, which estimates the asymmetry of the funnel plot. Positive values (Coef. > 0) indicate higher levels of effect size in studies with smaller sample sizes. [file 1741-7015-11-6-S7.PDF]

Additional File 7, Table S5: Egger test result bias for rs12979860, rs8099917 for SVR.

No. = number of studies; Coef.= asymmetry regression coefficient; Std.Err.= standard error; t= statistic; P>|t| = significance; and 95% CI= confidence interval.

Coef. corresponds to the intercept value in the regression equation, which estimates the asymmetry of funnel plot. Positive values (Coef.> 0) indicate higher levels of effect size in studies with smaller sample sizes.

Analysis	Polymorphism	No	Coef.	Std. Err.	t	P>|t|	95% CI	
SVR	rs12979860	42	-0.828	0.629	-1.32	0.196	-2.099	0.444
	rs8099917	39	1.744	0.587	2.97	0.005	0.554	2.933
