# Supplementary material for: Meta-analysis: implications of interleukin-28B polymorphisms in spontaneous and treatment-related clearance for patients with hepatitis C
Source: BMC Med. 2013 Jan 8;11:6. doi: 10.1186/1741-7015-11-6 (PMC3570369; doi:10.1186/1741-7015-11-6)
Supplement: Additional file 8 — Table S6, Genotype and allele frequencies stratified by ethnicity for all the polymorphisms included in the meta-analysis. (a) Sustained virologic response (SVR) and (b) spontaneous clearance (SC).Abbreviations: A, African; AA, African American; As, Asian; C, Caucasian; H, Hispanic; NA, North African; n.d., no data available. Favorable genotypes: rs12979860 (CC), rs8099917 (TT), rs12980275 (AA), rs8105790 (TT), rs11881222 (AA), rs8103142 (TT), rs7248668 (GG). There is conflicting information for rs10853728. [file 1741-7015-11-6-S8.PDF]

Additional File 8, Table S6. Genotype and allele frequencies stratified by ethnicity for all the polymorphisms included in the meta-analysis. a) SVR and b) SC.

Abbreviations: A= Africans; AA= African Americans; As= Asians; C= Caucasians; H= Hispanics; NA= North Africans; n.d., no data available.

Favourable genotypes: rs12979860 (CC), rs8099917 (TT), rs12980275 (AA), rs8105790 (TT), rs11881222 (AA), rs8103142 (TT), rs7248668 (GG); there is conflicting information for rs10853728.

a) SVR

		Genotype frequency			Allele frequency	
**SNP**	**Ethnicity**	**Homozyous A**	**Heterozygous**	**Homozygous B**	**Allele A**	**Allele B**
rs12979860	A (n=128)	0.234 (CC)	0.547 (CT)	0.219 (TT)	0.507	0.493
	AA (n= 473)	0.131	0.510	0.359	0.386	0.614
	As (n= 1880)	0.832	0.156	0.012	0.910	0.090
	C (n= 6031)	0.342	0.517	0.141	0.600	0.400
	H (n=215)	0.251	0.544	0.205	0.523	0.477
	NA (n= 60)	0.267	0.633	0.100	0.583	0.417
rs8099917	A (n= 129)	0.698 (TT)	0.279 (TG)	0.023 (GG)	0.837	0.163
	As (n= 4729)	0.752	0.232	0.016	0.868	0.132
	C (n= 3863)	0.562	0.387	0.051	0.755	0.245
	H (n=99)	0.293	0.576	0.131	0.581	0.419
rs12980275	As (n=1042)	0.802 (AA)	0.190 (AG)	0.008 (GG)	0.897	0.103
	C (n=1588)	0.342	0.520	0.138	0.602	0.398
	H (n=99)	0.263	0.576	0.162	0.551	0.449
rs8105790	As (n=728)	0.894 (TT)	0.104 (TC)	0.001 (CC)	0.946	0.054
	C (n=516)	0.500	0.432	0.068	0.716	0.284
rs11881222	As (n=728)	0.897 (AA)	0.102 (AG)	0.001 (GG)	0.948	0.052
	C (n=816)	0.338	0.529	0.132	0.603	0.397
rs8103142	As (n=728)	0 (TT)	0.999 (TC)	0.001 (CC)	0.499	0.501
	C (n=866)	0.194	0.612	0.194	0.500	0.500
rs7248668	As (n=728)	0.898 (GG)	0.100 (GA)	0.001 (AA)	0.948	0.051
	C (n=817)	0.520	0.424	0.056	0.732	0.268
rs10853728	As (n=728)	0.646 (CC)	0.320 (CG)	0.034 (GG)	0.806	0.194
	C (n=782)	0.373	0.425	0.202	0.585	0.415

b) SC

		Genotype frequency			Allele frequency	
**SNP**	**Ethnicity**	**Homozyous A**	**Heterozygous**	**Homozygous B**	**Allele A**	**Allele B**
rs12979860	C (n=1598)	0.458	0.451	0.091	0.683	0.316
	As (n=371)	0.485	0.509	0.005	0.739	0.259
rs8099917	C (n=1336)	0.586	0.366	0.047	0.769	0.231
	As (n=368)	0.867	0.133	0	0.933	0.066
