# Supplementary material for: Meta-analysis: implications of interleukin-28B polymorphisms in spontaneous and treatment-related clearance for patients with hepatitis C
Source: BMC Med. 2013 Jan 8;11:6. doi: 10.1186/1741-7015-11-6 (PMC3570369; doi:10.1186/1741-7015-11-6)
Supplement: Additional file 9 — Figure S3, Overall forest plot showing the association of rs12979860 with sustained virologic response (SVR). The vertical continuous line indicates no difference for SVR regarding IL28B genotype. The size of each square denotes the proportion of information provided by each trial. Pooled odds ratios were calculated from random-effects models with the DerSimonian-Laird method. (a) The number of patients with the favorable genotype (CC) who achieved SVR with respect to the total number of patients having the favorable genotype. (b) The number of patients with the unfavorable genotype (CT+TT) who achieved SVR with respect to the total number of patients having the unfavorable genotype. The dashed vertical red line represents overall OR. [file 1741-7015-11-6-S9.PDF]

Additional File 9, Figure S3: Overall forest plot showing the association of rs12979860 with SVR.

The vertical continuous line indicates no difference for SVR regarding IL28B genotype. The size of each square denotes the proportion of information provided by each trial. Pooled odds ratios were calculated from random-effect models with the DerSimonian-Laird method. a The number of patients with favourable genotype (CC) that achieved SVR with respect to the total number of patients showing favourable genotype. b The number of patients with unfavourable genotype (CT+TT) that achieved SVR with respect to the total number of patients showing unfavourable genotype. The dashed vertical red line represents overall OR.

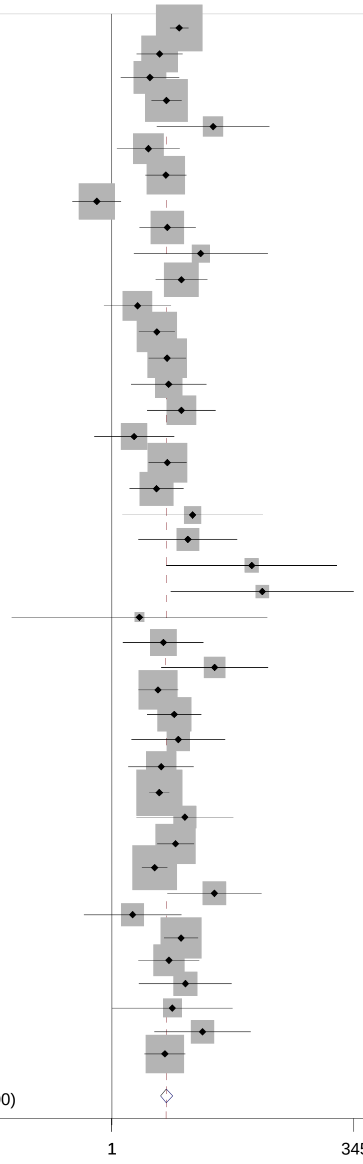

Study ID	OR (95% CI)	SVR, Favourable genotype [a]	SVR, Unfavourable genotype [b]	% Weight
Thompson et al (2010)	5.13 (4.09, 6.44)	340/512	299/1075	5.19
Montes-Cano et al (2010)	3.20 (1.84, 5.58)	68/102	45/117	3.23
Lotrich et al (2010)	2.53 (1.25, 5.13)	34/57	28/76	2.53
Stättermayer et al (2011)	3.77 (2.62, 5.44)	201/250	225/432	4.35
Dill et al (2011)	11.61 (2.98, 45.24)	16/19	17/54	0.99
Nattermann et al (2011)	2.44 (1.15, 5.22)	28/56	18/62	2.32
Chen et al (2011)	3.71 (2.26, 6.08)	521/654	38/74	3.57
Moghaddam et al (2011)	0.70 (0.39, 1.26)	99/128	127/153	3.05
Fattovich et al (2011)	3.87 (1.96, 7.62)	92/104	117/176	2.64
Liao et al (2011)	8.62 (1.71, 43.44)	56/82	2/10	0.73
Rallon et al (2011)	5.42 (2.89, 10.14)	65/86	40/110	2.87
Lindh et al (2011) 2	1.87 (0.83, 4.20)	56/66	75/100	2.13
Mangia et al (2011)	2.99 (1.94, 4.63)	89/128	141/326	3.91
de Rueda et al (2011)	3.84 (2.44, 6.04)	109/142	130/281	3.80
Lindh et al (2011) 1	3.98 (1.60, 9.91)	30/38	33/68	1.82
Ladero et al (2011)	5.41 (2.37, 12.36)	32/46	19/64	2.08
Pearlman et al (2011)	1.73 (0.66, 4.54)	10/27	16/63	1.68
O´Brien et al (2011)	3.86 (2.44, 6.12)	45/155	47/491	3.77
Halfon et al (2011)	2.95 (1.54, 5.67)	44/61	64/137	2.75
Lyoo et al (2011)	7.06 (1.29, 38.56)	40/57	2/8	0.67
Asselah et al (2011)	6.30 (1.90, 20.89)	18/22	25/60	1.21
Venegas et al (2011)	29.42 (3.75, 231.05)	19/20	31/79	0.47
Patel et al (2011)	38.00 (4.18, 345.04)	19/20	7/21	0.42
Chevaliez et al (2011)	1.96 (0.09, 42.97)	0/3	5/80	0.22
Sporea et al (2011)	3.49 (1.32, 9.23)	19/26	35/80	1.67
De Nicola et al (2012)	12.07 (3.31, 43.99)	21/24	29/79	1.07
Howell et al (2012)	3.08 (1.90, 5.01)	57/94	89/267	3.61
Cavalcante et al (2012)	4.55 (2.36, 8.75)	36/54	51/167	2.74
Payer et al (2012)	5.00 (1.61, 15.55)	21/26	21/46	1.32
Di Marco et al (2012)	3.31 (1.50, 7.30)	25/43	21/71	2.20
Fischer et al (2012)	3.16 (2.48, 4.04)	280/416	356/903	5.09
Younossi et al (2012)	5.86 (1.82, 18.89)	15/23	8/33	1.26
Vidal-Castiñeira et al (2012)	4.69 (3.00, 7.33)	111/149	99/258	3.85
Kobayashi et al (2012)	2.83 (2.09, 3.84)	520/772	94/223	4.73
Petta et al (2012)	11.98 (3.84, 37.39)	27/31	31/86	1.32
Tajir et al (2012)	1.67 (0.52, 5.38)	10/16	22/44	1.26
Valenti et al (2012)	5.35 (3.54, 8.10)	163/198	188/404	4.04
Karchava et al (2012)	3.99 (1.90, 8.37)	53/67	37/76	2.38
Tolmane et al (2012)	5.95 (1.94, 18.28)	34/38	50/85	1.34
Christensen et al (2012)	4.33 (1.01, 18.62)	14/17	14/27	0.88
Mira et al (2012)	9.00 (2.81, 28.78)	12/18	12/66	1.27
Amanzada et al (2012)	3.62 (2.20, 5.95)	67/104	67/201	3.55
**Overall** (I-squared = 52.3%, p = 0.000)	3.77 (3.25, 4.37)	3516/4951	2775/7233	100.00

NOTE: Weights are from random effects analysis
