# Supplementary material for: Meta-analysis: implications of interleukin-28B polymorphisms in spontaneous and treatment-related clearance for patients with hepatitis C
Source: BMC Med. 2013 Jan 8;11:6. doi: 10.1186/1741-7015-11-6 (PMC3570369; doi:10.1186/1741-7015-11-6)

Additional File 10, Figure S4: Forest plot showing the association between rs12979860 and SVR stratified by ethnicity.

See description in Supplemental Figure 3.

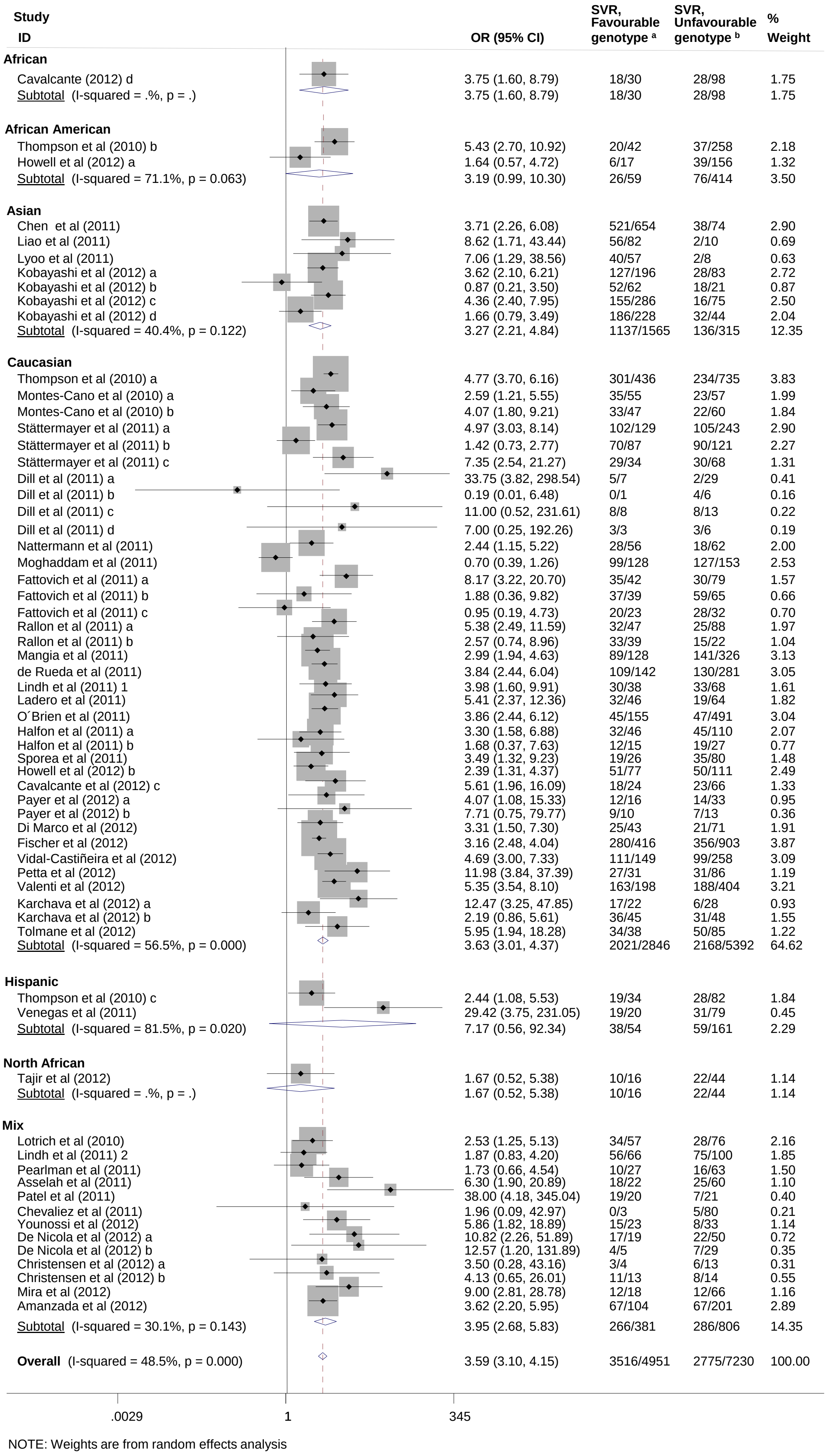

Supplement: Additional file 10 — Figure S4, Forest plot showing the association between rs12979860 and sustained virologic response (SVR) stratified by ethnicity. See description in Figure S3. [file 1741-7015-11-6-S10.PDF]
