# Supplementary material for: Meta-analysis: implications of interleukin-28B polymorphisms in spontaneous and treatment-related clearance for patients with hepatitis C
Source: BMC Med. 2013 Jan 8;11:6. doi: 10.1186/1741-7015-11-6 (PMC3570369; doi:10.1186/1741-7015-11-6)
Supplement: Additional file 11 — Figure S5, Forest plot showing the association between rs12979860 and sustained virologic response (SVR) stratified by hepatitis C virus (HCV) genotype. See description in Figure S3. [file 1741-7015-11-6-S11.PDF]

Additional File 11, Figure S5: Forest plot showing the association between rs12979860 and SVR stratified by HCV genotype.

See description in Supplemental Figure 3.

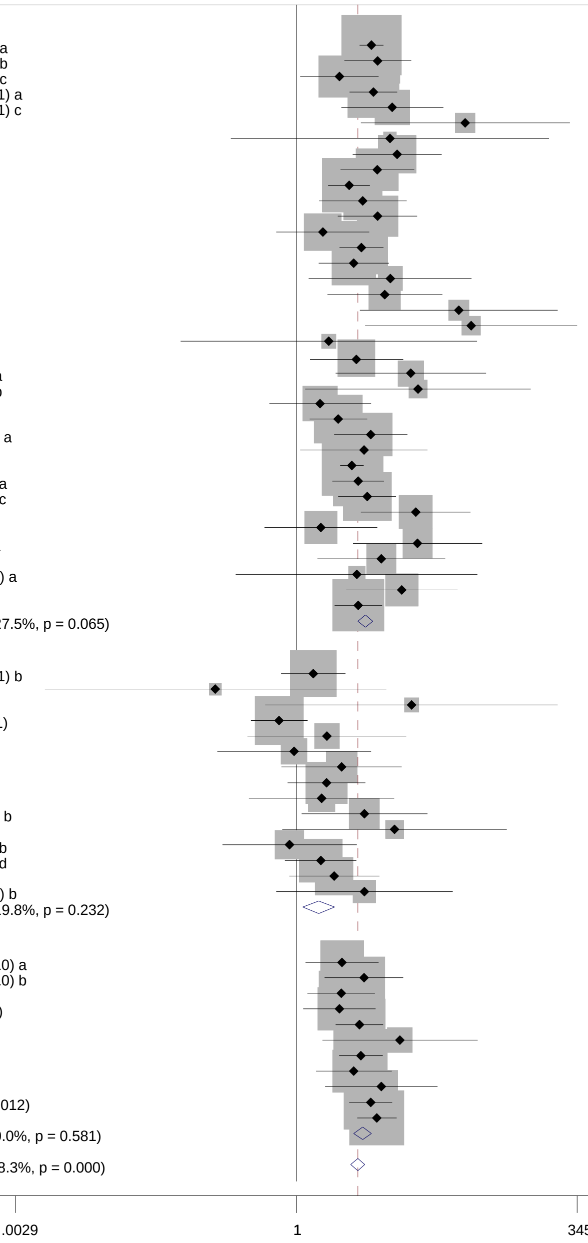

Study ID	OR (95% CI)	SVR, Favourable genotype [a]	SVR, Unfavourable genotype [b]	% Weight
**Genotype 1/4**				
Thompson et al (2010) a	4.77 (3.70, 6.16)	301/436	234/735	3.85
Thompson et al (2010) b	5.43 (2.70, 10.92)	20/42	37/258	2.18
Thompson et al (2010) c	2.44 (1.08, 5.53)	19/34	28/82	1.85
Stättermayer et al (2011) a	4.97 (3.03, 8.14)	102/129	105/243	2.91
Stättermayer et al (2011) c	7.35 (2.54, 21.27)	29/34	30/68	1.32
Dill et al (2011) a	33.75 (3.82, 298.54)	5/7	2/29	0.41
Dill et al (2011) d	7.00 (0.25, 192.26)	3/3	3/6	0.19
Fattovich et al (2011) a	8.17 (3.22, 20.70)	35/42	30/79	1.58
Rallon et al (2011) a	5.38 (2.49, 11.59)	32/47	25/88	1.98
Mangia et al (2011)	2.99 (1.94, 4.63)	89/128	141/326	3.15
Lindh et al (2011) 1	3.98 (1.60, 9.91)	30/38	33/68	1.61
Ladero et al (2011)	5.41 (2.37, 12.36)	32/46	19/64	1.82
Pearlman et al (2011)	1.73 (0.66, 4.54)	10/27	16/63	1.50
O´Brien et al (2011)	3.86 (2.44, 6.12)	45/155	47/491	3.05
Halfon et al (2011) a	3.30 (1.58, 6.88)	32/46	45/110	2.08
Lyoo et al (2011)	7.06 (1.29, 38.56)	40/57	2/8	0.63
Asselah et al (2011)	6.30 (1.90, 20.89)	18/22	25/60	1.11
Venegas et al (2011)	29.42 (3.75, 231.05)	19/20	31/79	0.45
Patel et al (2011)	38.00 (4.18, 345.04)	19/20	7/21	0.40
Chevaliez et al (2011)	1.96 (0.09, 42.97)	0/3	5/80	0.21
Sporea et al (2011)	3.49 (1.32, 9.23)	19/26	35/80	1.49
De Nicola et al (2012) a	10.82 (2.26, 51.89)	17/19	22/50	0.72
De Nicola et al (2012) b	12.57 (1.20, 131.89)	4/5	7/29	0.35
Howell et al (2012) a	1.64 (0.57, 4.72)	6/17	39/156	1.33
Howell et al (2012) b	2.39 (1.31, 4.37)	51/77	50/111	2.51
Cavalcante et al (2012) a	4.69 (2.19, 10.05)	25/39	35/127	2.00
Payer et al (2012) a	4.07 (1.08, 15.33)	12/16	14/33	0.95
Fischer et al (2012)	3.16 (2.48, 4.04)	280/416	356/903	3.89
Kobayashi et al (2012) a	3.62 (2.10, 6.21)	127/196	28/83	2.73
Kobayashi et al (2012) c	4.36 (2.40, 7.95)	155/286	16/75	2.52
Petta et al (2012)	11.98 (3.84, 37.39)	27/31	31/86	1.20
Tajir et al (2012)	1.67 (0.52, 5.38)	10/16	22/44	1.15
Karchava et al (2012) a	12.47 (3.25, 47.85)	17/22	6/28	0.93
Tolmane et al (2012) a	5.87 (1.55, 22.15)	16/19	30/63	0.95
Christensen et al (2012) a	3.50 (0.28, 43.16)	3/4	6/13	0.31
Mira et al (2012)	9.00 (2.81, 28.78)	12/18	12/66	1.16
Amanzada et al (2012)	3.62 (2.20, 5.95)	67/104	67/201	2.90
Subtotal (I-squared = 27.5%, p = 0.065)	4.20 (3.61, 4.90)	1728/2647	1641/5106	59.33
**Genotype 2/3**				
Stättermayer et al (2011) b	1.42 (0.73, 2.77)	70/87	90/121	2.28
Dill et al (2011) b	0.19 (0.01, 6.48)	0/1	4/6	0.16
Dill et al (2011) c	11.00 (0.52, 231.61)	8/8	8/13	0.22
Moghaddam et al (2011)	0.70 (0.39, 1.26)	99/128	127/153	2.55
Fattovich et al (2011) b	1.88 (0.36, 9.82)	37/39	59/65	0.66
Fattovich et al (2011) c	0.95 (0.19, 4.73)	20/23	28/32	0.70
Rallon et al (2011) b	2.57 (0.74, 8.96)	33/39	15/22	1.04
Lindh et al (2011) 2	1.87 (0.83, 4.20)	56/66	75/100	1.86
Halfon et al (2011) b	1.68 (0.37, 7.63)	12/15	19/27	0.77
Cavalcante et al (2012) b	4.13 (1.12, 15.25)	11/15	16/40	0.97
Payer et al (2012) b	7.71 (0.75, 79.77)	9/10	7/13	0.36
Kobayashi et al (2012) b	0.87 (0.21, 3.50)	52/62	18/21	0.87
Kobayashi et al (2012) d	1.66 (0.79, 3.49)	186/228	32/44	2.05
Karchava et al (2012) b	2.19 (0.86, 5.61)	36/45	31/48	1.55
Christensen et al (2012) b	4.13 (0.65, 26.01)	11/13	8/14	0.55
Subtotal (I-squared = 19.8%, p = 0.232)	1.59 (1.14, 2.22)	640/779	537/719	16.59
**Mix**				
Montes-Cano et al (2010) a	2.59 (1.21, 5.55)	35/55	23/57	1.99
Montes-Cano et al (2010) b	4.07 (1.80, 9.21)	33/47	22/60	1.85
Lotrich et al (2010)	2.53 (1.25, 5.13)	34/57	28/76	2.16
Nattermann et al (2011)	2.44 (1.15, 5.22)	28/56	18/62	2.01
Chen et al (2011)	3.71 (2.26, 6.08)	521/654	38/74	2.91
Liao et al (2011)	8.62 (1.71, 43.44)	56/82	2/10	0.69
de Rueda et al (2011)	3.84 (2.44, 6.04)	109/142	130/281	3.07
Di Marco et al (2012)	3.31 (1.50, 7.30)	25/43	21/71	1.91
Younossi et al (2012)	5.86 (1.82, 18.89)	15/23	8/33	1.15
Vidal-Castiñeira et al (2012)	4.69 (3.00, 7.33)	111/149	99/258	3.10
Valenti et al (2012)	5.35 (3.54, 8.10)	163/198	188/404	3.23
Subtotal (I-squared = 0.0%, p = 0.581)	3.97 (3.30, 4.77)	1130/1506	577/1386	24.07
**Overall** (I-squared = 48.3%, p = 0.000)	3.58 (3.10, 4.15)	3498/4932	2755/7211	100.00

NOTE: Weights are from random effects analysis
