# Supplementary material for: Meta-analysis: implications of interleukin-28B polymorphisms in spontaneous and treatment-related clearance for patients with hepatitis C
Source: BMC Med. 2013 Jan 8;11:6. doi: 10.1186/1741-7015-11-6 (PMC3570369; doi:10.1186/1741-7015-11-6)
Supplement: Additional file 12 — Table S7, Subgroup analysis by ethnicity and hepatitis C virus (HCV) genotype (pooled odds ratio and 95% confidence interval). ‡Data from only one study. * P < 0.05; ** P < 0.001. Abbreviations: A, African; AA, African American; As, Asian; C, Caucasian; H, Hispanic; n.d., no data available. [file 1741-7015-11-6-S12.PDF]

Additional File 12, Table S7: Subgroup analysis by ethnicity and HCV genotype. Pooled odds ratio and 95% confidence interval.

*Data from only one study
Abbreviations: A, Africans; AA, African Americans; As, Asians; C, Caucasians; H, Hispanics; n.d., no data available.

		HCV Genotype		
		1	2/3	4
**rs12979860**	**A**	1.667 (0.516-5.381)‡	n.d.	n.d.
	**AA**	3.191 (0.989-10.295)	n.d.	n.d.
	**As**	4.058 (2.745-5.999)**	1.439 (0.747-2.773)‡	n.d.
	**C**	4.174 (3.369-5.171)**	1.459 (0.922-2.310)	7.314 (2.658-20.123)**
	**H**	7.170 (0.557-92.338)	n.d.	n.d.
**rs8099917**	**AA**	n.d.	n.d.	n.d.
	**As**	5.708 (4.495-7.248)**	2.031 (1.185-3.483)*	n.d.
	**C**	3.637 (2.723-4.856)**	1.139 (0.769-1.686)	2.626 (1.233-5.594)*
	**H**	11.250 (3.515-36.002)‡**	n.d.	n.d.
