# Supplementary material for: Meta-analysis: implications of interleukin-28B polymorphisms in spontaneous and treatment-related clearance for patients with hepatitis C
Source: BMC Med. 2013 Jan 8;11:6. doi: 10.1186/1741-7015-11-6 (PMC3570369; doi:10.1186/1741-7015-11-6)
Supplement: Additional file 14 — Figure S7, Overall forest plot showing the association between rs8099917 and sustained virologic response (SVR). Superscripts: number of patients with (a) favorable genotype (TT) or (b) unfavorable genotype (TG+GG) who achieved SVR, with respect to the total number of patients with the favorable or unfavorable genotype, respectively. For extended details, see main description in Figure S3. [file 1741-7015-11-6-S14.PDF]

Additional File 14, Figure S7: Overall forest plot showing the association between rs8099917 and SVR.

Superscripts: number of patients with (a) favourable genotype (TT)/ (b) unfavourable genotype (TG+GG), that achieved SVR with respect to the total number of patients showing favourable / unfavourable genotype, respectively. For extended details see main description in Supplemental Figure 3.

Study ID	OR (95% CI)	SVR, Favourable genotype [a]	SVR, Unfavourable genotype [b]	% Weight
Tanaka et al (2009)	12.09 (6.54, 22.36)	125/196	15/118	3.06
Grebely et al (2010)	0.92 (0.30, 2.79)	18/29	16/25	1.81
Aparicio et al (2010)	4.77 (2.22, 10.24)	56/104	11/56	2.62
Stättermayer et al (2011)	2.39 (1.74, 3.29)	289/409	137/273	3.94
Dill et al (2011)	1.00 (0.13, 7.57)	5/8	5/8	0.76
Kurozaki et al (2011)	6.50 (3.88, 10.89)	173/346	20/150	3.37
Ochi et al (2011)	2.69 (2.16, 3.35)	840/1621	138/483	4.17
Yu et al (2011)	1.37 (0.58, 3.21)	386/432	43/50	2.38
Hsu et al (2011)	6.70 (1.94, 23.17)	67/77	7/14	1.58
Chen et al (2011)	3.10 (1.90, 5.06)	517/652	42/76	3.44
Sakamoto et al (2011)	3.01 (1.23, 7.34)	81/100	17/29	2.29
Moghaddam et al (2011)	0.93 (0.48, 1.80)	161/201	65/80	2.92
Fattovich et al (2011)	2.34 (1.35, 4.04)	129/158	80/122	3.26
Sinn et al (2011)	1.09 (0.36, 3.37)	74/100	13/18	1.78
Onomoto et al (2011)	19.20 (2.38, 155.05)	30/55	1/17	0.72
Lindh et al (2011) 1	7.18 (2.99, 17.22)	50/65	13/41	2.33
Ladero et al (2011)	2.84 (1.29, 6.27)	36/63	15/47	2.55
Hayashi et al (2011)	8.66 (4.34, 17.27)	127/219	11/80	2.83
Halfon et al (2011)	2.28 (1.28, 4.06)	73/116	35/82	3.17
Lyoo et al (2011)	4.59 (1.03, 20.53)	39/56	3/9	1.22
Venegas et al (2011)	11.25 (3.52, 36.00)	25/29	25/70	1.71
Miyaaki et al (2011)	4.51 (1.14, 17.74)	26/51	3/16	1.38
Miyamura et al (2011)	3.48 (1.14, 10.60)	24/53	5/26	1.80
Takita et al (2011)	7.21 (3.08, 16.87)	53/78	10/44	2.39
Huang et al (2012)	5.32 (2.44, 11.58)	121/187	10/39	2.58
Lange et al (2012)	2.76 (1.91, 4.00)	200/292	92/209	3.81
Cavalcante et al (2012)	2.51 (1.39, 4.52)	66/139	22/83	3.14
Di Marco (2012)	3.37 (1.47, 7.72)	35/68	11/46	2.45
Fischer et al (2012)	2.74 (2.19, 3.42)	413/692	220/627	4.17
Liu et al (2012)	9.18 (5.79, 14.55)	388/536	28/126	3.53
Miyase et al (2012)	5.15 (2.77, 9.57)	92/127	25/74	3.05
Li et al (2012)	5.90 (1.36, 25.66)	31/45	3/11	1.25
Inokuchi et al (2012)	8.84 (2.92, 26.76)	54/76	5/23	1.81
Kim et al (2012)	7.39 (1.92, 28.45)	34/57	3/18	1.41
Petta et al (2012)	6.72 (2.96, 15.26)	38/51	20/66	2.47
Saito et al (2012)	10.22 (5.16, 20.23)	80/130	13/96	2.86
Ogawa et al (2012)	4.38 (2.44, 7.83)	100/221	17/107	3.16
Amanzada et al (2012)	2.30 (1.45, 3.65)	80/147	54/158	3.53
Guo et al (2012)	3.99 (2.31, 6.88)	86/120	45/116	3.27
Overall (I-squared = 71.9%, p = 0.000)	3.86 (3.18, 4.69)	5222/8106	1298/3733	100.00

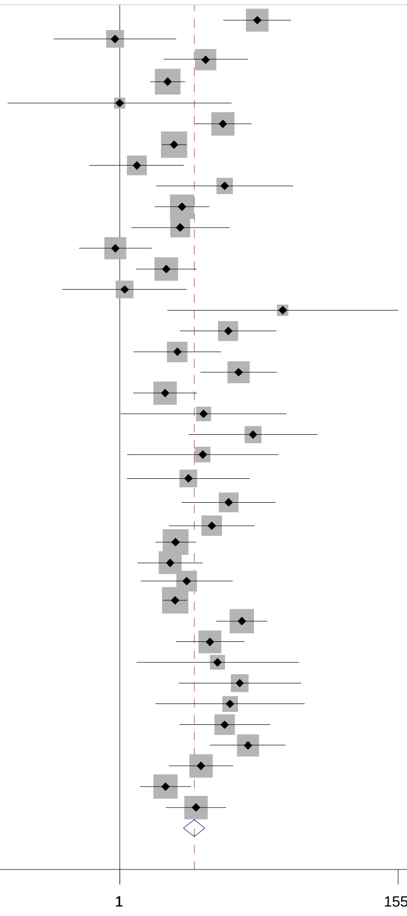

NOTE: Weights are from random effects analysis
