# Supplementary material for: Meta-analysis: implications of interleukin-28B polymorphisms in spontaneous and treatment-related clearance for patients with hepatitis C
Source: BMC Med. 2013 Jan 8;11:6. doi: 10.1186/1741-7015-11-6 (PMC3570369; doi:10.1186/1741-7015-11-6)
Supplement: Additional file 15 — Figure S8, Forest plot showing the association between rs8099917 and sustained virologic response (SVR), stratified by ethnicity. Superscripts: number of patients with (a) favorable genotype (TT) or (b) unfavorable genotype (TG+GG) who achieved SVR, with respect to the total number of patients having the favorable or unfavorable genotype, respectively. For complete details, see main description in Figure S3. [file 1741-7015-11-6-S15.PDF]

Additional File 15, Figure S8: Forest plot showing the association between rs8099917 and SVR stratified by ethnicity.

Superscripts: number of patients with (a) favourable genotype (TT)/ (b) unfavourable genotype (TG+GG), that achieved SVR with respect to the total number of patients showing favourable / unfavourable genotype, respectively. For complete details see main description in Supplemental Figure 3.

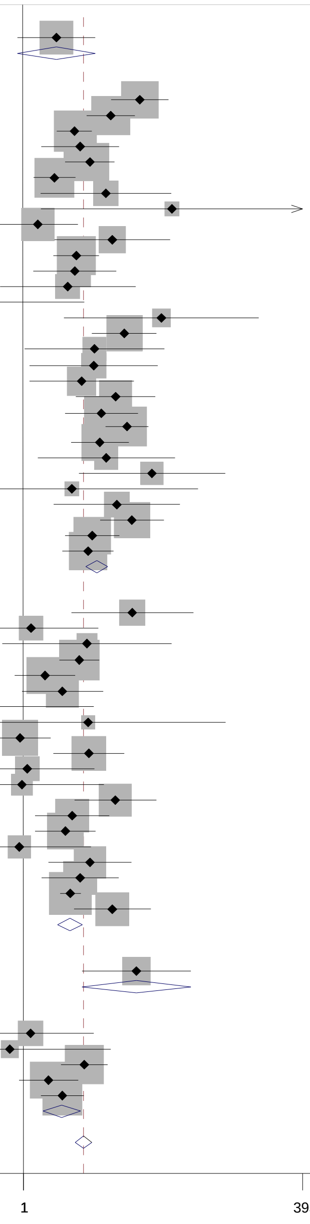

Study ID	OR (95% CI)	SVR, Favourable genotype [a]	SVR, Unfavourable genotype [b]	% Weight
**African**				
Cavalcante et al (2012) d	2.02 (0.88, 4.65)	37/90	10/39	1.98
Subtotal (I-squared = .%, p = .)	2.02 (0.88, 4.65)	37/90	10/39	1.98
**Asian**				
Tanaka et al (2009)	12.09 (6.54, 22.36)	125/196	15/118	2.47
Kurozaki et al (2011)	6.50 (3.88, 10.89)	173/346	20/150	2.70
Ochi et al (2011) a	2.98 (2.04, 4.34)	247/416	55/167	3.02
Ochi et al (2011) b	3.37 (1.46, 7.76)	59/140	8/45	1.98
Ochi et al (2011) c	4.15 (2.44, 7.06)	200/594	17/156	2.66
Ochi et al (2011) d	1.95 (1.24, 3.05)	289/415	53/98	2.86
Ochi et al (2011) e	5.83 (1.44, 23.61)	21/30	4/14	1.11
Ochi et al (2011) f	24.00 (1.46, 394.88)	24/26	1/3	0.36
Yu et al (2011)	1.37 (0.58, 3.21)	386/432	43/50	1.93
Hsu et al (2011)	6.70 (1.94, 23.17)	67/77	7/14	1.30
Chen et al (2011)	3.10 (1.90, 5.06)	517/652	42/76	2.76
Sakamoto et al (2011)	3.01 (1.23, 7.34)	81/100	17/29	1.86
Sinn et al (2011) a	2.58 (0.60, 11.04)	31/46	4/9	1.05
Sinn et al (2011) b	0.20 (0.01, 3.68)	43/54	9/9	0.34
Onomoto et al (2011)	19.20 (2.38, 155.05)	30/55	1/17	0.60
Hayashi et al (2011)	8.66 (4.34, 17.27)	127/219	11/80	2.29
Lyoo et al (2011)	4.59 (1.03, 20.53)	39/56	3/9	1.01
Miyaaki et al (2011)	4.51 (1.14, 17.74)	26/51	3/16	1.14
Miyamura et al (2011)	3.48 (1.14, 10.60)	24/53	5/26	1.47
Takita et al (2011)	7.21 (3.08, 16.87)	53/78	10/44	1.94
Huang et al (2012)	5.32 (2.44, 11.58)	121/187	10/39	2.09
Liu et al (2012)	9.18 (5.79, 14.55)	388/536	28/126	2.83
Miyase et al (2012)	5.15 (2.77, 9.57)	92/127	25/74	2.46
Li et al (2012)	5.90 (1.36, 25.66)	31/45	3/11	1.03
Inokuchi et al (2012) a	15.73 (3.29, 75.05)	37/57	2/19	0.95
Inokuchi et al (2012) b	2.83 (0.19, 41.99)	17/19	3/4	0.39
Kim et al (2012)	7.39 (1.92, 28.45)	34/57	3/18	1.16
Saito et al (2012)	10.22 (5.16, 20.23)	80/130	13/96	2.31
Ogawa et al (2012	4.38 (2.44, 7.83)	100/221	17/107	2.54
Guo et al (2012)	3.99 (2.31, 6.88)	86/120	45/116	2.63
Subtotal (I-squared = 60.3%, p = 0.000)	4.82 (3.80, 6.11)	3548/5535	477/1740	53.23
**Caucasian**				
Aparicio et al (2010) a	10.33 (2.81, 38.06)	26/52	3/34	1.22
Aparicio et al (2010) b	1.18 (0.28, 4.96)	23/36	6/10	1.07
Aparicio et al (2010) c	3.89 (0.64, 23.79)	7/16	2/12	0.76
Stättermayer et al (2011) a	3.32 (2.16, 5.11)	148/219	59/153	2.90
Stättermayer et al (2011) b	1.59 (0.83, 3.04)	95/118	65/90	2.39
Stättermayer et al (2011) c	2.31 (0.97, 5.51)	46/72	13/30	1.91
Dill et al (2011) b	0.17 (0.01, 4.51)	1/3	3/4	0.27
Dill et al (2011) d	4.00 (0.21, 75.66)	4/5	2/4	0.33
Moghaddam et al (2011)	0.93 (0.48, 1.80)	161/201	65/80	2.36
Fattovich et al (2011) a	4.05 (1.90, 8.65)	45/65	20/56	2.14
Fattovich et al (2011) b	1.09 (0.26, 4.60)	50/54	46/50	1.06
Fattovich et al (2011) c	0.97 (0.17, 5.61)	34/39	14/16	0.80
Lindh et al (2011) 1	7.18 (2.99, 17.22)	50/65	13/41	1.89
Ladero et al (2011)	2.84 (1.29, 6.27)	36/63	15/47	2.07
Halfon et al (2011) a	2.47 (1.29, 4.71)	51/86	26/70	2.39
Halfon et al (2011) b	0.92 (0.20, 4.26)	22/30	9/12	0.97
Cavalcante et al (2012) c	4.16 (1.71, 10.12)	29/47	12/43	1.87
Di Marco et al (2012)	3.37 (1.47, 7.72)	35/68	11/46	1.99
Fischer et al (2012)	2.74 (2.19, 3.42)	413/692	220/627	3.31
Petta et al (2012)	6.72 (2.96, 15.26)	38/51	20/66	2.01
Subtotal (I-squared = 50.8%, p = 0.005)	2.71 (2.07, 3.54)	1314/1982	624/1491	33.68
**Hispanic**				
Venegas et al (2011)	11.25 (3.52, 36.00)	25/29	25/70	1.40
Subtotal (I-squared = .%, p = .)	11.25 (3.52, 36.00)	25/29	25/70	1.40
**Mix**				
Grebely et al (2010) a	1.17 (0.30, 4.50)	14/23	8/14	1.16
Grebely et al (2010) b	0.75 (0.09, 6.47)	4/6	8/11	0.57
Lange et al (2012) a	3.68 (2.23, 6.08)	90/155	35/128	2.73
Lange et al (2012) b	1.72 (0.91, 3.24)	110/137	57/81	2.42
Amanzada et al (2012)	2.30 (1.45, 3.65)	80/147	54/158	2.83
Subtotal (I-squared = 34.4%, p = 0.192)	2.27 (1.52, 3.39)	298/468	162/392	9.71
**Overall** (I-squared = 64.0%, p = 0.000)	3.64 (3.04, 4.34)	5222/8104	1298/3732	100.00

NOTE: Weights are from random effects analysis
