# Supplementary material for: Meta-analysis: implications of interleukin-28B polymorphisms in spontaneous and treatment-related clearance for patients with hepatitis C
Source: BMC Med. 2013 Jan 8;11:6. doi: 10.1186/1741-7015-11-6 (PMC3570369; doi:10.1186/1741-7015-11-6)
Supplement: Additional file 18 — Figure S11, Overall forest plot showing the association between rs12980275 and sustained virologic response (SVR). Superscripts: number of patients with (a) favorable genotype (AA) or (b) unfavorable genotype (AG+GG) who achieved SVR, with respect to the total number of patients having the favorable or unfavorable genotype, respectively. For extended details, see main description in Figure S3. [file 1741-7015-11-6-S18.PDF]

Additional File 18, Figure S11: Overall forest plot showing the association between rs12980275 and SVR.

Superscripts: number of patients with (a) favourable genotype (AA)/ (b) unfavourable genotype (AG+GG), that achieved SVR with respect to the total number of patients showing favourable/unfavourable genotype, respectively. For extended details see main description in Supplemental Figure 3.

Study ID	OR (95% CI)	SVR, Favourable genotype [a]	SVR, Unfavourable genotype [b]	% Weight
Tanaka et al (2009)	8.83 (5.07, 15.39)	119/187	21/127	18.75
Grebely et al (2010)	1.46 (0.50, 4.24)	18/28	16/29	11.64
Chen et al (2011)	2.88 (1.77, 4.67)	514/649	45/79	19.83
Fattovich et al (2011)	4.99 (2.42, 10.26)	94/104	115/176	16.19
Venegas et al (2011)	8.84 (2.76, 28.34)	22/26	28/73	10.55
Fischer et al (2012)	2.61 (2.06, 3.31)	280/439	350/869	23.05
Overall (I-squared = 77.7%, p = 0.000)	3.95 (2.39, 6.53)	1047/1433	575/1353	100.00

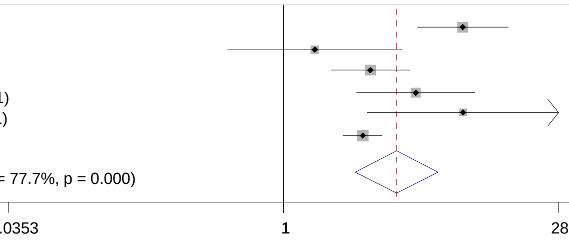

NOTE: Weights are from random effects analysis
