# Supplementary material for: Meta-analysis: implications of interleukin-28B polymorphisms in spontaneous and treatment-related clearance for patients with hepatitis C
Source: BMC Med. 2013 Jan 8;11:6. doi: 10.1186/1741-7015-11-6 (PMC3570369; doi:10.1186/1741-7015-11-6)
Supplement: Additional file 20 — Figure S13, Forest plot showing the association between rs12980275 and sustained virologic response (SVR) stratified by hepatitis C virus (HCV) genotype. Superscripts: number of patients with (a) favorable genotype (AA) or (b) unfavorable genotype (AG+GG) who achieved SVR, with respect to the total number of patients having the favorable or unfavorable genotype, respectively. For extended details, see main description in Figure S3. [file 1741-7015-11-6-S20.PDF]

Additional File 20, Figure S13: Forest plot showing the association between rs12980275 and SVR stratified by HCV genotype.

Superscripts: number of patients with (a) favourable genotype (AA)/ (b) unfavourable genotype (AG+GG), that achieved SVR with respect to the total number of patients showing favourable / unfavourable genotype, respectively. For extended details see main description in Supplemental Figure 3.

Study ID	OR (95% CI)	SVR, Favourable genotype [a]	SVR, Unfavourable genotype [b]	% Weight
**Genotype 1,4**				
Tanaka et al (2009)	8.83 (5.07, 15.39)	119/187	21/127	16.90
Fattovich et al (2011) a	10.34 (3.89, 27.50)	36/42	29/79	12.11
Venegas et al (2011)	8.84 (2.76, 28.34)	22/26	28/73	10.30
Fischer et al (2012)	2.61 (2.06, 3.31)	280/439	350/869	19.97
Subtotal (I-squared = 87.4%, p = 0.000)	6.33 (2.64, 15.16)	457/694	428/1148	59.27
**Genotype 2,3**				
Fattovich et al (2011) b	4.59 (0.54, 38.78)	38/39	58/65	4.70
Fattovich et al (2011) c	0.95 (0.19, 4.73)	20/23	28/32	7.09
Subtotal (I-squared = 27.3%, p = 0.241)	1.78 (0.38, 8.25)	58/62	86/97	11.79
**Mix**				
Grebely et al (2010)	1.46 (0.50, 4.24)	18/28	16/29	11.24
Chen et al (2011)	2.88 (1.77, 4.67)	514/649	45/79	17.70
Subtotal (I-squared = 22.5%, p = 0.256)	2.44 (1.38, 4.32)	532/677	61/108	28.93
**Overall** (I-squared = 75.7%, p = 0.000)	3.92 (2.31, 6.63)	1047/1433	575/1353	100.00

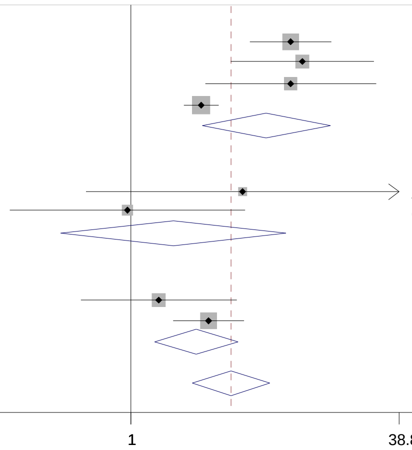

NOTE: Weights are from random effects analysis
