# Supplementary material for: Meta-analysis: implications of interleukin-28B polymorphisms in spontaneous and treatment-related clearance for patients with hepatitis C
Source: BMC Med. 2013 Jan 8;11:6. doi: 10.1186/1741-7015-11-6 (PMC3570369; doi:10.1186/1741-7015-11-6)

**Additional File 22, Figure S15: Overall forest plot showing the association between rs7248668 and SVR.**

Pooled odds ratios were calculated from fixed-effect models with the Mantel-Haenszel method. Superscripts: number of patients with (a) favourable genotype (GG)/ (b) unfavourable genotype (GA+AA), that achieved SVR with respect to the total number of patients showing favourable/unfavourable genotype, respectively For extended details see main description in Supplemental Figure 3.

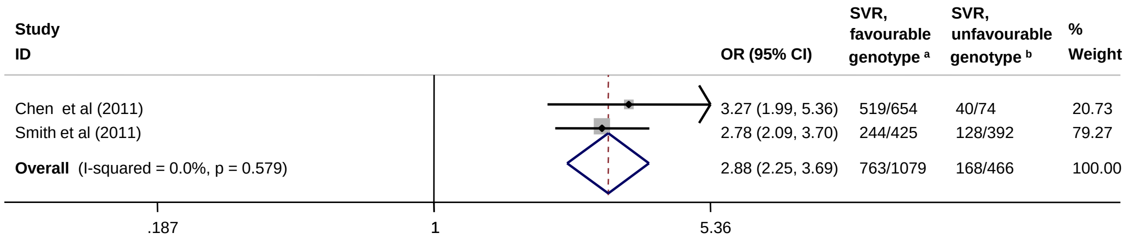

Supplement: Additional file 22 — Figure S15, Overall forest plot showing the association between rs7248668 and sustained virologic response (SVR). Pooled odds ratios were calculated from fixed-effect models with the Mantel-Haenszel method. Superscripts: number of patients with (a) favorable genotype (GG) or (b) unfavorable genotype (GA+AA) who achieved SVR, with respect to the total number of patients having the favorable or unfavorable genotype, respectively For extended details, see main description in Figure S3. [file 1741-7015-11-6-S22.PDF]
