# Supplementary material for: Meta-analysis: implications of interleukin-28B polymorphisms in spontaneous and treatment-related clearance for patients with hepatitis C
Source: BMC Med. 2013 Jan 8;11:6. doi: 10.1186/1741-7015-11-6 (PMC3570369; doi:10.1186/1741-7015-11-6)

Additional File 24, Figure S17: Overall forest plot showing the association between rs8105790 and SVR.

Pooled odds ratios were calculated from fixed-effect models with the Mantel-Haenszel method. Superscripts: number of patients with (a) favourable genotype (TT)/ (b) unfavourable genotype (TC+CC), that achieved SVR with respect to the total number of patients showing favourable/unfavourable genotype, respectively. For extended details see main description in Supplemental Figure 3.

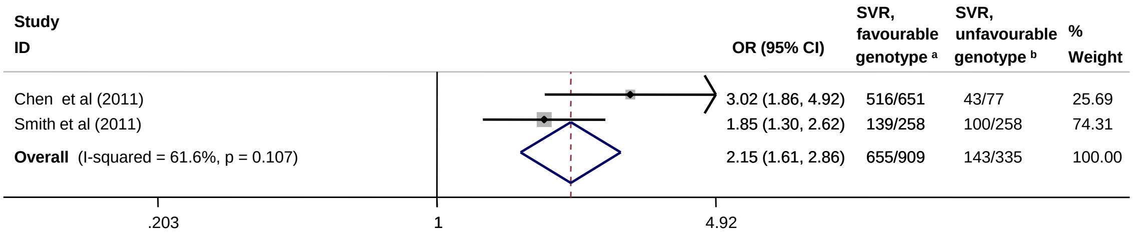

Supplement: Additional file 24 — Figure S17, Overall forest plot showing the association between rs8105790 and sustained virologic response (SVR). Pooled odds ratios were calculated from fixed-effect models with the Mantel-Haenszel method. Superscripts: number of patients with (a) favorable genotype (TT) or (b) unfavorable genotype (TC+CC) who achieved SVR, with respect to the total number of patients having the favorable or unfavorable genotype, respectively. For extended details, see main description in Figure S3. [file 1741-7015-11-6-S24.PDF]
