# Supplementary material for: Meta-analysis: implications of interleukin-28B polymorphisms in spontaneous and treatment-related clearance for patients with hepatitis C
Source: BMC Med. 2013 Jan 8;11:6. doi: 10.1186/1741-7015-11-6 (PMC3570369; doi:10.1186/1741-7015-11-6)
Supplement: Additional file 25 — Figure S18, Overall forest plot showing the association between rs10853728 and sustained virologic response (SVR). Superscripts: number of patients with (a) favorable genotype (CC) or (b) unfavorable genotype (CG+GG) who achieved SVR, with respect to the total number of patients having the favorable or unfavorable genotype, respectively. For extended details, see main description in Figure S3. [file 1741-7015-11-6-S25.PDF]

Additional File 25, Figure S18: Overall forest plot showing the association between rs10853728 and SVR.

Superscripts: number of patients with (a) favourable genotype (CC)/ (b) unfavourable genotype (CG+GG), that achieved SVR with respect to the total number of patients showing favourable / unfavourable genotype, respectively. For extended details see main description in Supplemental Figure 3.

Study ID	OR (95% CI)	SVR, favourable genotype [a]	SVR, unfavourable genotype [b]	% Weight
Yu et al (2011)	0.82 (0.44, 1.53)	281/318	148/164	14.51
Chen et al (2011)	1.69 (1.19, 2.40)	377/470	182/258	29.69
Smith et al (2011)	1.08 (0.81, 1.44)	136/292	219/490	55.80
**Overall** (I-squared = 64.3%, p = 0.061)	1.20 (0.82, 1.76)	794/1080	549/912	100.00

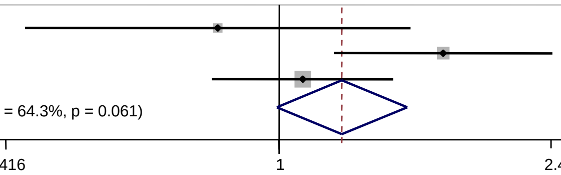

NOTE: Weights are from random effects analysis
