# Supplementary material for: Meta-analysis: implications of interleukin-28B polymorphisms in spontaneous and treatment-related clearance for patients with hepatitis C
Source: BMC Med. 2013 Jan 8;11:6. doi: 10.1186/1741-7015-11-6 (PMC3570369; doi:10.1186/1741-7015-11-6)

Additional File 26, Figure S19: Forest plot showing the associations between IL28B polymorphisms reported in only one study and SVR.

Superscripts: number of patients with (a) favourable genotype (CC)/ (b) unfavourable genotype (CG+GG), that achieved SVR with respect to the total number of patients showing favourable / unfavourable genotype, respectively. * SNPs from Smith et al article; † SNPs from Chen et al article.

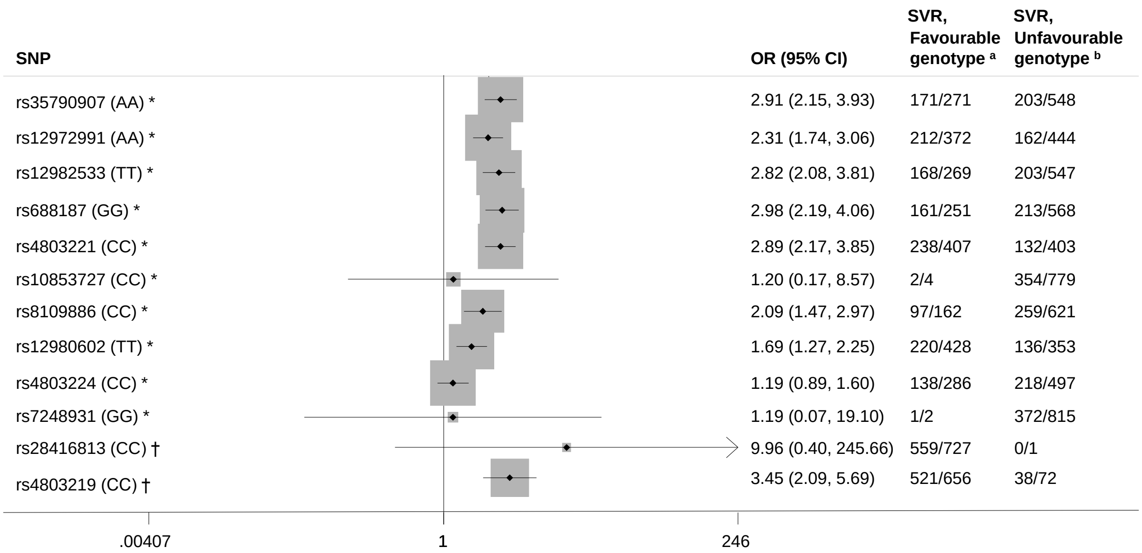

Supplement: Additional file 26 — Figure S19, Forest plot showing the associations between interleukin 28B (IL28B) gene polymorphisms reported in only one study and sustained virologic response (SVR). Superscripts: number of patients with (a) favorable genotype (CC) or (b) unfavorable genotype (CG+GG) who achieved SVR with respect to the total number of patients having the favorable or unfavorable genotype, respectively. * Single-nucleotide polymorphisms (SNPs) from Smith et al article; † SNPs from Chen et al article. [file 1741-7015-11-6-S26.PDF]
