# Supplementary material for: Meta-analysis: implications of interleukin-28B polymorphisms in spontaneous and treatment-related clearance for patients with hepatitis C
Source: BMC Med. 2013 Jan 8;11:6. doi: 10.1186/1741-7015-11-6 (PMC3570369; doi:10.1186/1741-7015-11-6)
Supplement: Additional file 31 — Figure S24, Forest plot showing the association between rs12980275 and spontaneous clearance (SC). The vertical continuous line indicates no difference in SC for the interleukin 28B IL28B) genotype. Pooled odds ratios were calculated from random-effects models with the DerSimonian-Laird method. (a) The number of patients with the favorable genotype who achieved SC, with respect to the total number of patients having the favorable genotype. (b) The number of patients with the unfavorable genotype who achieved SC, with respect to the total number of patients having the unfavorable genotype. For complete details, see main description in Figure 3. [file 1741-7015-11-6-S31.PDF]

Additional File 31, Figure S24: Forest plot showing the association between rs12980275 and SC.

The vertical continuous line indicates no difference for SC regarding IL28B genotype. Pooled odds ratios were calculated from random-effect models with the DerSimonian-Laird method. a The number of patients with favourable genotype that achieved SC with respect to the total number of patients showing favourable genotype. b The number of patients with unfavourable genotype that achieve SC with respect to the total number of patients showing unfavourable genotype. For complete details see main description in Figure 3.

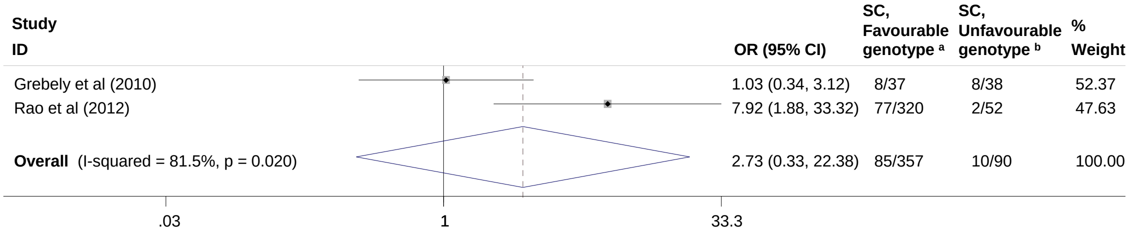

NOTE: Weights are from random effects analysis
