# Supplementary material for: Meta-analysis: implications of interleukin-28B polymorphisms in spontaneous and treatment-related clearance for patients with hepatitis C
Source: BMC Med. 2013 Jan 8;11:6. doi: 10.1186/1741-7015-11-6 (PMC3570369; doi:10.1186/1741-7015-11-6)

Additional File 32, Figure S25: Forest plot showing the associations between IL28B polymorphisms reported in only one study and SC.

For extended details see main description in Supplemental Figure 3.
*SNPs from Rao et al. ; †SNPs from Renda et al.

Study ID	OR (95% CI)	SC, Favourable genotype [a]	SC, Unfavourable genotype [b]	% Weight
rs10853728 (CC) *	2.32 (1.22, 4.42)	67/269	13/104	46.95
rs11881222 (AA) *	1.91 (0.69, 5.26)	6/18	73/352	15.79
rs12980275 (AA) *	7.92 (1.88, 33.32)	77/320	2/52	8.71
rs4803219 (CC) *	8.38 (0.49, 142.06)	80/358	0/14	2.48
rs4803223 (AA) *	2.57 (0.88, 7.45)	73/329	4/40	18.51
rs8105790 (TT) *	14.88 (2.02, 109.72)	77/315	1/47	4.38
rs8103142 (TT) †	13.50 (3.07, 59.46)	15/19	5/23	3.18

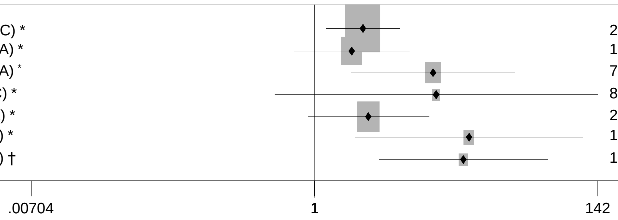

Supplement: Additional file 32 — Figure S25, Forest plot showing the associations between interleukin 28B (IL28B) gene polymorphisms reported in only one study and spontaneous clearance (SC). For extended details, see main description in Figure S3. [file 1741-7015-11-6-S32.PDF]
